# Generalized Lagrangian Path Approach to Manifestly-Covariant Quantum Gravity Theory

**DOI:** 10.3390/e20030205

**Published:** 2018-03-19

**Authors:** Massimo Tessarotto, Claudio Cremaschini

**Affiliations:** 1 Department of Mathematics and Geosciences, University of Trieste, Via Valerio 12, 34127 Trieste, Italy; 2 Institute of Physics, Faculty of Philosophy and Science, Silesian University in Opava, Bezručovo nám.13, CZ-74601 Opava, Czech Republic; 3 Institute of Physics and Research Center for Theoretical Physics and Astrophysics, Faculty of Philosophy and Science, Silesian University in Opava, Bezručovo nám.13, CZ-74601 Opava, Czech Republic

**Keywords:** quantum mechanics, generalized Lagrangian paths, covariant quantum gravity, emergent space-time, Gaussian-like solutions, 03.65.Ca, 03.65.Ta

## Abstract

A trajectory-based representation for the quantum theory of the gravitational field is formulated. This is achieved in terms of a covariant Generalized Lagrangian-Path (GLP) approach which relies on a suitable statistical representation of Bohmian Lagrangian trajectories, referred to here as *GLP-representation*. The result is established in the framework of the manifestly-covariant quantum gravity theory (CQG-theory) proposed recently and the related CQG-wave equation advancing in proper-time the quantum state associated with massive gravitons. Generally non-stationary analytical solutions for the CQG-wave equation with non-vanishing cosmological constant are determined in such a framework, which exhibit Gaussian-like probability densities that are non-dispersive in proper-time. As a remarkable outcome of the theory achieved by implementing these analytical solutions, the existence of an emergent gravity phenomenon is proven to hold. Accordingly, it is shown that a mean-field background space-time metric tensor can be expressed in terms of a suitable statistical average of stochastic fluctuations of the quantum gravitational field whose quantum-wave dynamics is described by GLP trajectories.

## 1. Introduction

The search for a theory of quantum gravity that is consistent both with the principles of quantum mechanics [1] as well as with the postulates of the classical Einstein theory of General Relativity (GR) [2,3,4] has represented so far one of the most challenging and hard-to-solve conceptual problems of mathematical and theoretical physics alike. The crucial issue is about the possibility of achieving in the context of either classical or quantum relativistic theories, and in particular for a quantum theory of gravity, a truly coordinate- (i.e., frame-) independent representation, namely which satisfies, besides the general covariance principle, also the so-called principle of manifest covariance. In fact, although the choice of special coordinate systems is always legitimate for all physical systems either discrete or continuous, including in particular classical and quantum gravity, the intrinsic objective nature of physical laws makes them frame-independent.

However, for these principles to actually apply, a background space-time picture must hold. This means, more precisely, that a suitable classical curved space-time $\left\{Q^{4},\hat{g}\right\}$ must exist with respect to which both general covariance principle and principle of manifest covariance can be prescribed. As a consequence, when parameterized with respect to a coordinate system $r\equiv \left\{r^{\mu }\right\}$ the same space-time must be endowed with a well-defined (i.e., uniquely prescribed and hence deterministic) symmetric metric tensor $\hat{g},$ represented equivalently in terms of its covariant $\hat{g}\equiv \left\{{\hat{g}}_{\mu \nu }\right\}$ and countervariant $\hat{g}\equiv \left\{{\hat{g}}^{\mu \nu }\right\}$ forms , which is referred to in the following as the “*background*” field tensor. In particular, $Q^{4}$ can be identified with a time-oriented four dimensional Riemann space-time. Thus, although the precise choice of the same background space-time itself remains in principle arbitrary, as a consequence of the principle of manifest covariance it should always be possible to represent all quantum observables (of the theory), including the corresponding quantum Hamiltonian operator and quantum canonical variables/operators (see below), in 4-tensor form. This requires to cast them exclusively as 4-tensor fields with respect to the group of local point transformations (LPT group)

(1) $$r\equiv \left\{r^{\mu }\right\}\to r^{\prime }\equiv \left\{{r^{\prime }}^{\mu }\right\}=r^{\prime }\left(r\right)$$

mapping $\left\{Q^{4},\hat{g}\right\}$ in itself [5].

In such a framework $\hat{g}$ is considered as a classical (i.e., deterministic) tensor field, to be identified as the metric tensor field of $\left\{Q^{4},\hat{g}\right\}$ which—as such—determines the geometric properties of the same space-time. This means more precisely that:

*Prescription a:* Its covariant and countervariant components, i.e., respectively, ${\hat{g}}_{\mu \nu }$ and ${\hat{g}}^{{}_{\mu \nu }}$, must lower and raise tensor indices of arbitrary tensor fields and also prescribe the standard connections (Christoffel symbols) appearing in the covariant derivatives.

*Prescription b:* It determines the Ricci tensor, the Ricci 4-scalar and the coupling contained in the stress–energy tensor due to external sources, in the sequel, respectively, identified with the symbols ${\hat{R}}_{\mu \nu }$ $\equiv R_{\mu \nu }\left(\hat{g}\right)$, $\hat{R}$ $\equiv R\left(\hat{g}\right)\equiv {\hat{g}}^{\alpha \beta }{\hat{R}}_{\alpha \beta }$ and ${\hat{T}}_{\mu \nu }=T_{\mu \nu }\left(\hat{g}\right)$.

*Prescription c:* Consequently, $\hat{g}$ can be identified with a particular solution of the Einstein field equations

(2) $${\hat{R}}_{\mu \nu }-\frac{1}{2}\left[\hat{R}-2\Lambda \right]{\hat{g}}_{\mu \nu }=\frac{8\pi G}{c^{4}}{\hat{T}}_{\mu \nu },$$

where as usual Λ denotes the cosmological constant.

*Prescription d:* $\hat{g}$ determines uniquely *the Riemann distance s*, or *proper-time*, on the space-time $\left\{Q^{4},\hat{g}\right\}$ by means of the 4-scalar equation

(3) $$ds^{2}={\hat{g}}_{\mu \nu }\left(r,s\right)dr^{\mu }dr^{\nu }.$$

One notices that, in accordance with [5], here $dr^{\mu }\equiv dr^{\mu }\left(s\right)$ and ds identify, respectively, the 4-tensor displacement and its corresponding 4-scalar line-element (arc length), both evaluated along a suitable worldline. For this purpose, the latter is identified with an arbitrary geodetics $r\left(s\right)\equiv \left\{r^{\mu }\left(s\right)\right\}$ belonging to $\left\{Q^{4},\hat{g}\right\}$ that crosses an arbitrary 4-position $r^{\mu }\equiv r_{o}^{\mu }$, and hence fulfills the initial condition $r^{\mu }\left(s_{o}\right)=r_{o}^{\mu }$, at some proper time $s_{o}$ (which for definiteness can always be set $s_{o}=0$). Consequently, the definition of proper time remains unambiguous and unique also for arbitrary finite values of s∈I (with I≡R the real axis), being identified with the arc length along the (unique) geodetics $r\left(s\right)\equiv \left\{r^{\mu }\left(s\right)\right\}$ joining $r^{\mu }\left(s_{o}\right)=r_{o}^{\mu }$ with an arbitrary 4-position $r_{1}^{\mu },$ i.e., such that $r^{\mu }\left(s_{1}\right)=r_{1}^{\mu }$ for a given $s_{1}$ is assumed to exist. For example, the proper time can always be defined along an appropriate observer geodetics.

*Prescription e:* One notices that in principle the background metric tensor might be taken of the form $\hat{g}\left(r,s\right)\equiv \left\{{\hat{g}}_{\mu \nu }\left(r,s\right)\right\}$, i.e., allowed to depend explicitly also on the proper time s. In the following, however, we shall restrict the treatment to the customary case in which the background metric tensor solution of the Einstein field equations is purely dependent only on the 4-position $r^{\mu }$, namely is of the form

(4) $$\hat{g}=\hat{g}\left(r\right),$$

which identifies a *stationary metric tensor*.

Next, let us consider the prescription holding for the Lagrangian continuum coordinates $g\equiv \left\{g^{\mu \nu }\right\}$ and the conjugate momentum operator $\pi \equiv \left\{\pi^{\mu \nu }\right\}$, again both to be considered as 4-tensor fields with respect to the group of local point transformations in Equation (1):

*Prescription f:* As a consequence of the stationarity assumption in Equation (4), for all sets $\left(r,s\right)\in \left\{Q^{4},\hat{g}\right\}\times I$ tensor decompositions of the form

(5) $$\left\{\begin{array}{c} g\left(r,s\right)=\hat{g}\left(r\right)+\delta g\left(r,s\right),\\ \pi (r,s)=\delta \pi (r,s), \end{array}\right.$$

will be assumed to hold for the quantum gravity theory, with $\delta g\left(r,s\right)\equiv \left\{\delta g_{\mu \nu }\left(r,s\right)\right\}$ and $\delta \pi \left(r,s\right)\equiv \left\{\delta \pi_{\mu \nu }\left(r,s\right)\right\}$ denoting the corresponding *quantum fluctuations*, represented by a coordinate displacement field and momentum operator which by assumption may depend explicitly on the variables (r,s).

A promising new scenario for quantum gravity fulfilling these requirements has recently been established in [6,7,8,9,10,11]. This is realized by the theory of manifestly-covariant quantum gravity, denoted as CQG-theory, which is based on the manifestly-covariant canonical quantization (*g-quantization*) of the classical Hamiltonian state $\left\{g(r,s),\pi (r,s)\right\}$. It must be clarified that in the present treatment the concept of manifest covariance means that CQG-theory is realized by a formulation in which all classical and quantum Hamiltonian field variables or operators, including continuum coordinates, conjugate momenta and Hamiltonian densities transform as 4-tensors, i.e., fulfill covariance tensor transformation laws with respect to the group of local point transformations in Equation (1). Although a manifestly-covariant theory of this type need not necessarily be unique, the involved notion of manifest covariance given here is certainly unambiguously determined when the background space-time $\left\{Q^{4},\hat{g}\right\}$ is prescribed. On the other hand, an alternative route is also available. This is based on the preliminary introduction of a non-canonical mapping in which the classical (and hence also the quantum) Hamiltonian state $\left\{g(r,s),\pi (r,s)\right\}$ is mapped by means of a diffeomorphism onto a suitable set of non-canonical variables

(6) $$\left\{g(r,s),\pi (r,s)\right\}\Leftrightarrow \left\{\eta (r,s),\chi (r,s)\right\},$$

in which, however, $\eta \left(r,s\right)\equiv \left\{\eta_{\alpha \beta }\left(r,s\right)\right\}$ and $\chi \left(r,s\right)\equiv \left\{\chi_{\alpha \beta }\left(r,s\right)\right\}$ are not represented by 4-tensor variables. When expressed in terms of the transformed variables $\left\{\eta (r,s),\chi (r,s)\right\}$ CQG-theory does not lose obviously the property of covariance (its equations remain covariant with respect to the LPT-group) although its variables (i.e., $\left\{\eta (r,s),\chi (r,s)\right\}$) are not represented by 4-tensors. Such a notion will be referred to as property of plain covariance of the theory. The distinction between the two notions of covariance (manifest or plain) is, however, important. In fact manifest covariance represents a stronger condition for the realization of a quantum theory of gravitational field with respect to literature approaches which, instead, may or may not rely on weaker notions of covariance such as that of plain covariance (see also subsequent discussion in Section 2).

As such, CQG-theory is endowed with a number of key features, since: A) it preserves the background metric tensor $\hat{g}\left(r\right)$ which is identified with a classical field tensor; B) it satisfies the *quantum unitarity principle*, i.e., the quantum probability is conserved; C) it is *constraint-free*, in the sense that the quantum Lagrangian variables g≡g(r,s) are identified with independent tensor fields; D) it is *non-perturbative* so that the quantum fluctuations δg(r,s) and δπ(r,s) need not be regarded as asymptotically “small” in some appropriate sense with respect to the background metric tensor $\hat{g}\left(r\right)$. Its foundations (for a detailed discussion see [9]) lie on the preliminary establishment of a variational formulation of GR achieved in the context of a covariant DeDonder–Weyl-type approach to continuum field-Hamiltonian dynamics [12,13,14,15,16,17,18,19] in which the background space-time $\left\{Q^{4},\hat{g}\right\}$ is considered prescribed [7,8].

In the following, we intend to shed further light on key aspects of the CQG-theory which are intimately related with its consistent realization. These include in particular two crucial “*tests of consistency*” for CQG-theory which should actually be regarded as mandatory physical prerequisites for any quantum theory of gravity fulfilling both the principles of general and manifest covariance.

The first one is that, although quantum corrections may in principle occur [7,8], it must be possible to preserve the functional form of the Einstein field equations consistent with the so-called *emergent gravity picture*. More precisely, the latter equations should follow uniquely from quantum theory itself *without performing the semiclassical continuum limit* (namely obtained letting in particular ℏ→0; see for example [20] where the derivation of the Einstein field equation was discussed in the context of loop quantum gravity). This property will be referred to here as ”*first-type emergent-gravity paradigm*”.

The second test of consistency, to be investigated here, refers instead to the validity of an emergent-gravity picture also for the deterministic background metric tensor $\hat{g}\left(r\right),$ in the sense that the same $\hat{g}\left(r\right)$ should be prescribed by means of a suitably-defined quantum/stochastic expectation value of the quantum state. This property will be denoted here as “*second-type emergent-gravity paradigm*”. A basic requirement needed for its verification is manifestly the determination of a suitable class of particular solutions of the quantum wave-function, i.e., the CQG-wave equation for the quantum state ψ(g,r,s) earlier pointed out in [10].

With this hindsight in mind, in the following Eulerian and Lagrangian representations are preliminarily distinguished for the CQG-wave equation and its corresponding set of quantum hydrodynamic equations (QHE). The latter are implied by the Madelung representation [21] of the quantum wave function written in Eulerian form $\psi \equiv \psi \left(g,r,s\right)$, namely distinguishing the dependences in terms of the Lagrangian coordinates $g\equiv \left\{g_{\mu \nu }\right\}$ and the parameters (r,s) as

(7) $$\psi \left(g,r,s\right)=\sqrt{\rho (g,r,s)}\exp\left\{\frac{i}{\hbar }S^{\left(q\right)}\left(g,r,s\right)\right\}.$$

Here, the real fields $\left\{\rho ,S^{\left(q\right)}\right\}\equiv \left\{\rho \left(g,r,s\right)={\left|\psi (g,r,s)\right|}^{2},S^{\left(q\right)}\left(g,r,s\right)\right\}$ identify the quantum fluid 4-scalar fields written in Eulerian form, namely the quantum probability density function (PDF) and the quantum phase-function. In particular, the intent of the investigation concerns the introduction of a trajectory-based or Lagrangian representation of CQG-theory (see subsequent Section 4 and Section 5), to be distinguished from the Eulerian one (see Section 3) and referred to here as *Generalized Lagrangian-path approach* to CQG-theory. This goal is obtained by means of an appropriate parameterization of the corresponding set of quantum hydrodynamic equations, following in turn from the CQG wave-equation and based on the Madelung representation recalled above (see Equation (7)). More precisely, this concerns the investigation of:

- *Goal #1*: Explicit solutions of the CQG-quantum hydrodynamic equations satisfying suitable physical requirements.
- *Goal #2*: The “emergent” character of the classical background space-time metric tensor $\hat{g}\left(r\right)$, to be determined in terms of quantum theory. Accordingly, the background metric tensor $\hat{g}\left(r\right)$ should be identified with a suitably-defined quantum expectation value of the quantum state, i.e., weighted in terms of the corresponding quantum probability density (PDF).
- *Goal #3*: The existence of either stationary or, more generally, non-stationary solutions with respect to the proper-time *s*, i.e., explicitly dependent on *s*, for the quantum state ψ expressed via the Madelung representation (see Equation (7)).
- *Goal #4*: The search of Gaussian-like or Gaussian realizations for the quantum PDF ρ.
- *Goal #5*: The search of separable solutions of the quantum Hamilton-Jacobi (H-J) equation in terms of the quantum phase-function $S^{\left(q\right)}$ and the investigation of their qualitative properties and in particular their asymptotic behavior for s→+∞.

For the tasks indicated above, in close similarity with non-relativistic quantum mechanics (see [22,23]), two choices are in principle available. The first one is based on the introduction of deterministic Lagrangian trajectories $\left\{g(s),s\in I\right\}$, or Lagrangian-Paths (LP), analogous to those adopted in the context of the Bohmian representation of non-relativistic quantum mechanics [24,25,26,27,28,29,30,31]. This provides a Bohmian interpretation (of CQG-theory) which is ontologically equivalent to CQG-theory itself [32].

Hence, the tensor field $\delta g\left(s\right)\equiv \delta g_{L}\left(s\right)$ is uniquely determined by means of a map of the type

(8) $$s\to \delta g_{L}\left(s\right)\equiv \delta g_{L}\left(r\left(s\right),s\right),$$

with r=r(s) denoting the parameterization in terms of geodetic curves associated with the classical background field tensor $\hat{g}\left(r\right)\equiv \hat{g}\left(r\left(s\right)\right)$ (see *Prescription d* above, [9] and related discussion in Section 4), so that, in terms of $g_{L}\left(s\right)\equiv g\left(s\right)$, it follows that $\left\{g(s),s\in I\right\}\equiv \left\{g_{L}\left(s\right)=\hat{g}\left(r\left(s\right)\right)+\delta g_{L}\left(s\right),s\in I\right\}$. The second choice, instead, and the one which is at the basis of the GLP trajectory-based approach (or *GLP-representation*) adopted here, is achieved in terms of suitable stochastic, i.e., intrinsically non-unique, Lagrangian trajectories which are referred to here as *Generalized Lagrangian Paths* (GLP). Such a notion, which is inspired and extends to CQG-theory the analogous approach earlier developed for non-relativistic quantum mechanics [22], is based on a suitable generalization of the concept of LP (see Section 5 below). In such a context, each deterministic LP $\left\{g(s),s\in I\right\}$ is replaced with a continuous statistical ensemble of *stochastic GLP trajectories* $\left\{G(s),s\in I\right\}$. More precisely, introducing in analogy with Equation (5) the tensor decomposition

(9) $$G\left(s\right)=\hat{g}\left(r\left(s\right)\right)+\delta G\left(s\right),$$

with $\delta G\left(s\right)\equiv \left\{\delta G_{\mu \nu }\left(r\left(s\right),s\right)\right\}$ being a suitable tensor field denoted as *GLP-displacement* to be later defined, each GLP trajectory

(10) $$\left\{G(s),s\in I\right\}\equiv \left\{\hat{g}\left(r\left(s\right)\right)+\delta G\left(s\right),s\in I\right\}$$

is parameterized in terms of the displacement field, to be considered as a stochastic field tensor,

(11) Δg=δg(s)−δG(s),

with $\Delta g\equiv \left\{\Delta g_{\mu \nu }\right\}$ denoting a suitable constant second-order tensor field referred to here as *stochastic displacement field tensor*. For definiteness, it is required that its covariant components at proper-times *s* and $s_{o}$, $\Delta g_{\mu \nu }\left(s\right)=\delta g_{\mu \nu }\left(s\right)-\delta G_{\mu \nu }\left(s\right)$ and $\Delta g_{\mu \nu }\left(s_{o}\right)=\delta g_{\mu \nu }\left(s_{o}\right)-\delta G_{\mu \nu }\left(s_{o}\right)$, are prescribed so that for all $s,s_{o}\in I$

(12) $$\Delta g_{\mu \nu }\left(s\right)=\Delta g_{\mu \nu }\left(s_{o}\right).$$

Then, this implies that its counter-variant components $\Delta g^{\mu \nu }\left(s\right)$ and $\Delta g^{\mu \nu }\left(s_{o}\right)$ can be equivalently determined in terms of the prescribed field tensors ${\hat{g}}_{\mu \nu }\left(r\right)\equiv \hat{g}\left(r\left(s\right)\right)$ or ${\hat{g}}_{\mu \nu }\left(r_{o}\right)\equiv \hat{g}\left(r\left(s_{o}\right)\right)$, so that one also has necessarily for all $s,s_{o}\in I$ that:

(13) $$\Delta g^{\mu \nu }\left(s\right)=\Delta g^{\mu \nu }\left(s_{o}\right).$$

Consequently, each GLP trajectory is actually represented by a configuration-space curve of the type $\left\{G(s),s\in I\right\}\equiv \left\{\hat{g}\left(r\left(s\right)\right)+\delta g\left(s\right)-\Delta g,s\in I\right\},$ so that upon varying the stochastic displacement field tensor Δg it actually defines a statistical ensemble of trajectories. In terms of them, i.e., by parameterizing the CQG wave-function ψ(g,r,s) (or equivalently the corresponding quantum fluid fields) in terms of the GLP-displacement δG(s)=δg(s)−Δg, the *GLP-representation* of CQG-theory is then achieved. This amounts to introduce the composed mapping $\psi \left(g,r,s\right)\to \psi (G\left(s\right),\Delta g,\hat{g},r,s)$, where $\psi (G\left(s\right),\Delta g,\hat{g},r,s)$ denotes the *GLP-parameterized quantum wave-function* in which the dependence in terms of the displacement tensor field Δg is explicitly allowed.

As shown in Section 5, the adoption of the GLP parameterization for CQG-theory actually leaves unchanged the underlying axioms established in [10], thus providing a Lagrangian representation of CQG-theory which is ontologically equivalent to CQG-theory itself. The remarkable new aspects of the GLP formalism, however, are that it will be shown: First, to determine a solution method for the CGQ-wave equation, to be referred to here as *GLP-approach*, permitting the explicit construction of physically-relevant particular realizations of the CGQ-quantum state $\psi \left(s\right)$. Second, to realize quantum solutions which are consistent with the emergent-gravity picture. In particular, for this purpose, the background field tensor will be shown to be determined equivalently either in terms of quantum expectation values or via a suitably-prescribed stochastic average of the quantum field tensor $g_{\mu \nu }$. This includes the determination of particular solutions of the CQG-wave equation which, consistent with Goal #1, satisfy the following physical requirements:

- *Requirement #1:* the quantum wave-function ψ(s) is *dynamically consistent*, namely for which the PDF $\rho \left(g,r,s\right)\equiv {\left|\psi (g,r,s)\right|}^{2}$ associated with the quantum wave-function ψ(g,r,s) is globally prescribed and summable in the quantum configuration space $U_{g}$ in such a way that the corresponding probability ${\left|\psi \right|}^{2}d\left(g\right)$ is similarly globally conserved for arbitrary subsets of the quantum configuration space $U_{g}$. As discussed below a prerequisite for meeting such a requirement is the validity of suitable Heisenberg inequalities earlier determined in [11].
- *Requirement #2:*ψ(s) exhibits the *explicit dependence in terms of a stochastic observable*, so to yield a so-called Stochastic-Variable Approach to quantum theory [22,33,34,35]. In the context of CQG-theory this should be generally identified with a 4-tensor field depending on the physical quantum observable $g_{\mu \nu }\left(r,s\right)$ and realizing a stochastic variable endowed with a stochastic probability density, i.e., dependent on a suitable stochastic field. Such a stochastic field will be identified in the following with the second-order real and observable stochastic displacement field tensor $\Delta g=\left\{\Delta g_{\mu \nu }\right\}$ defined by Equation (11) which by assumption depends functionally on $g_{\mu \nu }$ (and hence $\delta g_{\mu \nu }\left(r,s\right)$ too).
- *Requirement #3:* the PDF ρ is endowed with a *Gaussian-like* behavior and is *non-dispersive* in character, namely in the sense of assuming that in the subset of the proper-time axis *I* in which ψ is defined, its probability density ${\left|\psi \right|}^{2}$ can be identified for all s∈I≡R with a Gaussian-like PDF depending on Δg and $\hat{g}$, and thus by itself realizes a stochastic function. These particular solutions of the CQG-wave equation are generally non-stationary and are required to preserve their Gaussian-like character, and therefore to be non-dispersive, i.e., free of any spreading behavior during the proper-time quantum dynamical evolution.
- *Requirement #4:* the quantum wave function holds for arbitrary realizations of the deterministic background metric tensor $\hat{g}\left(r\right)$ and in particular in the case of vacuum solutions of the Einstein field equations.

Requirements #1–#4 are physically motivated. More precisely, the first one is needed to warrant the validity of the quantum unitarity principle, i.e., the conservation of quantum probability. The second requirement, instead, is instrumental for the present theory. In fact, as clarified below, the existence of the stochastic tensor observable Δg(g) is mandatory for the development of a GLP-approach in the context of CQG-theory. The third requirement is related to the issue about the physical origin of the cosmological constant [36]. The existence of Gaussian-like solutions for the quantum PDF $\rho \left(s\right)$ is mandatory in order to establish the connection between the CQG-theory and the Einstein field equations and to identify its precise quantum origin in terms of the Bohm vacuum interaction [32,37,38]. Finally, Requirement #4 is intimately related to the principle of manifest covariance and the deterministic character of the background metric tensor $\hat{g}\left(r\right)$.

As a further remark, one notices that Requirements #2 and #3 are qualitatively similar to those set at the basis of the GLP-approach developed for non-relativistic quantum mechanics. These led to the identification and proof of existence of non-dispersive Gaussian-like, or even properly Gaussian, particular solutions of the Schroedinger equation originally conjectured by Schrödinger himself in 1926 [39]. It is therefore natural to conjecture that analogous properties should hold in the context of the CQG-theory. As a remarkable conceptual outcome of the GLP theory, it is then shown that the discovery of analytical solutions satisfying physical Requirements #1–#4 allows for the investigation of theoretical aspects of the quantization of the gravitational field which go beyond the framework of so-called first-quantization, toward inclusion of second-quantization effects. This refers to quantum interactions of the gravitational field with itself which are intrinsically proper-time dependent contributions generated by the quantum wave dynamics retained in the solution of the same background metric tensor. In particular, in this work the existence of an emergent gravity phenomenon is displayed, which establishes a precise relationship between the background metric tensor ${\hat{g}}_{\mu \nu }$ and the quantum field $g_{\mu \nu }$. In detail, it is shown that ${\hat{g}}_{\mu \nu }$ can be represented as a mean-field background space-time metric tensor provided by a statistical moment of the Gaussian (or more generally Gaussian-like) PDF ρ. Hence, from the physical point of view ${\hat{g}}_{\mu \nu }$ can be effectively interpreted as arising from a statistical average of stochastic fluctuations of the quantum gravitational field $g_{\mu \nu }$ whose quantum-wave dynamics is described by GLP trajectories.

In detail, the structure of the paper is as follows. First, a qualitative comparison between CQG-theory and literature approaches to quantum gravity and its Bohmian formulation is proposed in Section 2. The Eulerian representation of CQG-theory is then presented in Section 3. Subsequently, the Lagrangian-path and Generalized Lagrangian-path representations are pointed out in Section 4 and Section 5, together with their Bohmian, i.e., deterministic, and correspondingly stochastic interpretations. Next, consistent with the axioms of CQG-theory, in Section 6, the establishment of the stochastic probability density attached with the stochastic displacement field tensor Δg is achieved. This is shown to be necessarily identified with the initial quantum PDF. In connection with such a prescription, in the same section the problem is posed of the construction of generalized Gaussian particular solutions for the quantum PDF ρ(g,r,s). Subsequently, in Section 7 the search of separable solutions of the corresponding quantum H-J equation is investigated. As a result, asymptotic conditions are investigated warranting the quantum phase function to be expressed in terms of polynomials of Δg. Finally, in Section 8, the main conclusions of the paper are drawn, while Appendix A and Appendix B contain mathematical details of the calculations.

## 2. Quantum Gravity Theories and Bohmian Formulation in Literature

This section is intended to provide a summary of the relevant conceptual features of CQG theory, together with an exhaustive discussion of literature works dealing with quantum gravity theories and corresponding Bohmian formulations. The aim of such a comparison with previous literature is twofold. From one side, we intend pointing out the main differences and significant progresses of CQG-theory from alternative approaches to quantum gravity. From the other side, we are interested in stating which are the common aspects of the present approach with other quantum theories of the gravitational field, and in which sense CQG-theory and the literature formulations discussed here can be reconciled or regarded as complementary. A review of the mathematical foundations of CQG theory and its Hamiltonian structure is treated separately in Section 3.

We start by noting that, according to [40] quantization methods, in both quantum mechanics and quantum gravity, can be classified in two classes, denoted, respectively, as the canonical and the covariant approaches. These differ in the way in which both the quantum state and the space-time are treated. In fact, the canonical quantization approach is based, first on the preliminary introduction of (3 + 1) or (2 + 2)-decompositions (or foliations [41,42,43]) for the representation of the space-time and, second, on the adoption of a quantum state represented in terms of non-4-tensor continuum fields. As such, by construction these theories are not covariant with respect to the LPT-group (1). Nevertheless, they still may retain well-definite covariance properties with respect to appropriate subgroups of local point transformations. For example, in the case of the (3 + 1)-decomposition covariance is warranted with respect to arbitrary point transformations which preserve the same foliation. In the covariant approaches, instead, typically all physical quantities including the quantum state are represented exclusively by means of 4-tensor fields. so that the property of manifest covariance remains fulfilled. Consequently, for these approaches, covariant quantization involves the assumption of some sort of classical background space-time structure on which a quantum gravity theory is constructed, for example identified with the flat Minkowski space-time. To realize such a strategy, however, it turns out that the quantum state is typically represented in terms of superabundant variables. Thus, in such cases, covariant quantization may also require the treatment of suitable constraint conditions.

Let us briefly analyze both approaches in more detail, considering first the canonical approach. A choice of this type is exemplified by the one adopted by Dirac and based on the Dirac constrained dynamics [44,45,46,47,48]. Dirac Hamiltonian approach to quantum gravity is not manifestly covariant, in reference both to transformation properties with respect to local as well as non-local point transformations (see discussion in [5]). In this picture in fact the field variable is identified with the metric tensor $g_{\mu \nu }$, but the corresponding “generalized velocity” is defined as $g_{\mu \nu ,0}$, namely with respect to the “time” component of the 4-position. This choice necessarily violates the principle of manifest covariance [7,8]. Consequently, in Dirac’s canonical theory, the canonical momentum remains identified with the manifestly non-tensorial quantity $\pi_{Dirac}^{\mu \nu }=\frac{\partial L_{EH}}{\partial g_{\mu \nu ,0}}$, where $L_{EH}$ is the Einstein-Hilbert variational Lagrangian density.

The same kind of ingredients is at the basis of the approach developed by Arnowitt, Deser and Misner (ADM theory, 1959–1962 [49]). In addition, in the ADM case, manifest covariance is lost because of the adoption of Lagrangian and Hamiltonian variables which are not 4-tensors. In fact, ADM theory is based on the introduction of a (3 + 1)-decomposition of space-time which, by construction, is foliation dependent, in the sense that it relies on a peculiar choice of a family of GR frames for which “time” and “space” transform separately, so that space-time is effectively split into the direct product of a one-dimensional time and a three-dimensional space subsets, respectively [50]. A quantum gravity theory constructed upon the ADM Hamiltonian formulation of gravitational field leads to postulating a quantum wave equation of Wheeler-DeWitt type [51]. The latter one is expressed as an evolution Schrödinger-like equation advancing the dynamics of the wave function with respect to the coordinate-time *t* of the ADM foliation, which is not an invariant parameter. In addition, in the absence of background space-time, the same equation carries a conceptual problem related in principle to the definition of the same coordinate time, which is simultaneously the dynamical parameter and a component of space-time which must be quantized by solving the wave equation. This marks a point of difference with respect to CQG theory and CQG-wave equation (see Equation (16) below), which represents a dynamical evolution equation with respect to an invariant (i.e., 4-scalar) proper-time *s* defined on the prescribed background space-time, without introduction of any kind of space-time foliation.

Another important approach is the one exemplified by the choice of so-called Ashtekar variables, originally identified respectively with a suitable self-dual spinorial connection (the generalized coordinates) and their conjugate momenta (see [52,53]). Ashtekar variables provide an alternative canonical representation of General Relativity, and this choice is at the basis of the so-called “loop representation of quantum general relativity” [54] usually referred to as “loop quantum gravity” (LQG) and first introduced by Rovelli and Smolin during 1988–1990 [55,56] (see also [57]). Nevertheless, the Ashtekar variables can also be shown to be by construction intrinsically manifestly non-tensorial in character. The basic consequence is that also the canonical representation of Einstein field equations based on these variables, as well as ultimately also LQG itself, violates the principle of manifest covariance. In contrast, in the framework of CQG-theory the choice of Hamiltonian state and quantum variables satisfies manifest covariance, whereby the dynamical variables are expressed by means of 4-tensor quantities.

However, despite these considerations, it must be stressed that both the canonical approach and CQG-theory can be regarded also complementary from a certain point of view, this because they exhibit distinctive physical properties associated with two canonical Hamiltonian structures underlying General Relativity itself. The corresponding Hamiltonian flows, however, are different, being referred to an appropriate coordinate-time of space-time foliation in the canonical approach, and to a suitable invariant proper-time in the present theory. Consequently, the physical interpretation of quantum theories of General Relativity build upon these Hamiltonian structures remain distinctive. The CQG-theory in fact reveals the possible existence of a discrete spectrum of metric tensors having non-vanishing momenta at quantum level, while canonical approaches deal with the quantum discretization of single space-time hypersurfaces implied by space-time foliation.

Let us now consider the covariant approaches to quantum gravity [58,59,60]. In this case, the usual strategy is to split the space-time metric tensor $g_{\mu \nu }$ in two parts according to the decomposition of the type $g_{\mu \nu }=\eta_{\mu \nu }+h_{\mu \nu }$, where $\eta_{\mu \nu }$ is the background metric tensor defining the space-time geometry (usually identified with the flat background), and $h_{\mu \nu }$ is the dynamical field (deviation field) for which quantization applies. From the conceptual point of view there are some similarities between the literature covariant approaches and the manifestly-covariant quantum gravity theory adopted here. The main points of contact are: (1) the adoption of 4-tensor variables, without invoking any space-time foliation; (2) the implementation of a first-quantization approach, in the sense that there exists by assumption a continuum classical background space-time with a geometric connotation, over which the relevant quantum fields are dynamically evolving; and (3) the adoption of superabundant variables, which in the two approaches are identified with the sets ($\eta_{\mu \nu },h_{\mu \nu }$) and (${\hat{g}}_{\mu \nu },g_{\mu \nu }$) respectively.

It is important nevertheless to emphasize the relevant differences existing with respect to literature covariant approaches. First, CQG-theory is intrinsically non-perturbative in character, so that the background metric tensor can be identified with an arbitrary continuum solution of the Einstein equations (not necessarily the flat space-time), while a priori the canonical variable $g_{\mu \nu }$ is not required to be necessarily a perturbation field. On the other hand, a decomposition of the type in Equation (5) resembling the one invoked in covariant literature approaches can always be introduced a posteriori for the implementation of appropriate analytical solution methods, like GLP theory proposed here or the analytical evaluation of discrete-spectrum quantum solutions discussed in [10]. Second, the present theory is constructed starting from the DeDonder–Weyl manifestly-covariant approach [12,13]. Consequently, CQG-theory is based on a variational formulation which relies on the introduction of a synchronous variational principle for the Einstein equations first reported in [7]. This represents a unique feature of manifestly-covariant quantum gravity theory, since previous literature is actually based on the adoption of asynchronous variational principles, i.e., in which the invariant volume element is considered variational rather than prescribed. As shown in [7], it is precisely the synchronous principle which allows the distinction between variational and extremal (or prescribed) metric tensors, and the consequent introduction of non-vanishing canonical momenta. The same feature has also made possible the formulation of manifestly-covariant classical Lagrangian, Hamiltonian and Hamilton–Jacobi theories of General Relativity and the corresponding subsequent manifestly-covariant quantum theory. Third, in CQG-theory superabundant unconstrained variables are implemented, while the same covariant quantization holds with respect to a four dimensional space-time, with no extra-dimensions being required for its prescription.

Finally, regarding covariant quantization, a further interesting comparison concerns the Batalin-Vilkovisky formalism originally developed in [61,62,63,64]. This method is usually implemented for the quantization of gauge field theories and topological field theories in Lagrangian formulation [65,66,67], while the corresponding Hamiltonian formulation can be found in [61]. Further critical aspects of the Batalin-Vilkovisky formalism can be found for example in [68]. In the case of the gravitational field it has been formerly applied in the context of perturbative quantum gravity to treat constraints arising from initial metric decomposition (i.e., in reference with the so-called gauge-fixing and ghost terms). Its basic features are the adoption of an asynchronous Lagrangian variational principle of General Relativity [7], the use of superabundant canonical variables and the consequent introduction of constraints. These features mark the main differences with CQG-theory, which is non-perturbative, constraint-free and follows from the synchronous Lagrangian variational principle defined in [7].

In view of these considerations, CQG-theory can be said to realize at the same time both a canonical and a manifestly-covariant quantization method, in this way establishing a connection with former canonical and covariant approaches. Nevertheless, a number of conceptual new features of the present theory depart in several ways from previous literature. This conclusion is supported by the analytical results already established by CQG-theory and presented in [10,11], which concern the existence of invariant discrete-energy spectrum for the quantum gravitational field, the graviton mass estimate associated with a non-vanishing cosmological constant and the validity of Heisenberg inequalities.

Extending further these results, in the following a trajectory-based representation of CQG-theory is developed, which permits the analytical construction of generally non-stationary solutions of the CQG-wave equation. Previous efforts to construct Bohmian representations of canonical quantum gravity and applications to cosmology have been pursued in the past literature. These are typically based on the Wheeler–DeWitt quantum equation. Relevant progresses in this directions can be found for example in [69,70,71,72], where conceptual features/differences characterizing the Bohmian approach to quantum gravity (in terms of trajectories) with respect to previous customary approaches were clearly stated. The GLP representation of CQG-theory proposed in the present paper shares the conceptual advantages of adopting a Bohmian approach to quantum physics. On the other hand, it differs from the mentioned literature in that it is built upon CQG-theory, which is manifestly covariant contrary to the Wheeler–DeWitt equation, and more important because it has a stochastic character, namely in the sense that single Bohmian trajectories are replaced by ensembles of stochastic trajectories with prescribed probability density.

## 3. Eulerian Representation

In this section, the basic formalism of CQG-theory formulated in [9,10] is recalled. Starting point of CQG-theory is the realization of the quantum-wave function which, for an arbitrary prescribed background space-time $\left(Q^{4},\hat{g}\right)$, determines the CQG-quantum state. In analogy with non-relativistic quantum mechanics, this can be first prescribed in the so-called Eulerian form. In this picture, the state is assumed to to depend on two sets of independent variables, respectively, represented by suitable configuration-space Lagrangian variables and, second, by the space-time coordinates and time. In the present case these are identified with the continuum field variables (Lagrangian coordinates) $g\equiv \left\{g_{\mu \nu }\right\}$ and, respectively, by the 4-position $r\equiv \{r^{\mu }\}$ and the background space-time proper-time *s*, so that the wave function takes generally the form ψ≡ψ(g,r,s), where a possible explicit dependence in terms of the background metric tensor $\hat{g}$ is understood. Regarding the notations, first $g=\left\{g_{\mu \nu }\right\}$ spans the quantum configuration space $U_{g}$ of the same wave-function, i.e., the set on which the associated quantum PDF $\rho \left(g,r,s\right)={\left|\psi (g,r,s)\right|}^{2}$ is prescribed. Second, $g=\left\{g_{\mu \nu }\right\}$ is realized by means of real symmetric tensors, so that $U_{g}$ is a 10-dimensional real vector space, namely $U_{g}\subseteq R^{10}$. Third, in the whole time-axis I≡R, $r\equiv \left\{r^{\mu }\right\}$ denotes the instantaneous 4-position of suitably-prescribed space-time trajectories r=r(s),while the explicit s-dependence includes also the possible dependence (of ψ) in terms of the corresponding tangent 4-vector, i.e., $t\left(s\right)\equiv \left\{t^{\mu }\left(s\right)\right\}\equiv \frac{dr^{\mu }\left(s\right)}{ds}$. Here, $\frac{d}{ds}$ identifies the total covariant *s*-derivative operator

(14) $$\frac{d}{ds}\equiv {\left.\frac{d}{ds}\right|}_{r}+{\left.\frac{d}{ds}\right|}_{s},$$

with ${\left.\frac{d}{ds}\right|}_{r}\equiv {\left.\frac{\partial }{\partial s}\right|}_{r}$ and ${\left.\frac{d}{ds}\right|}_{s}\equiv t^{\alpha }\nabla_{\alpha }$ being the covariant *s*-derivatives performed at constant $r\equiv \left\{r^{\mu }\right\}$ and constant *s* respectively.

A realization of the parameterization ψ≡ψ(g,r,s) is provided by the geodetics of the metric field tensor $\hat{g}\equiv \hat{g}\left(r\right)$, namely the integral curves of the initial-value problem [9,10]

(15) $$\left\{\begin{array}{c} \frac{dr^{\mu }\left(s\right)}{ds}=t^{\mu }\left(s\right),\\ \frac{Dt^{\mu }\left(s\right)}{Ds}=0,\\ r^{\mu }\left(s_{o}\right)=r_{o}^{\mu },\\ t^{\mu }\left(s_{o}\right)=t_{o}^{\mu }, \end{array}\right.$$

with $\left(r_{o}^{\mu },t_{o}^{\mu }\right)$ denoting, respectively, arbitrary initial 4-position of $\left\{Q^{4},\hat{g}\right\}$ and a corresponding (arbitrary) tangent 4-vector, while the standard connections in the covariant derivative $\frac{D}{Ds}$ are prescribed again in terms the background metric tensor $\hat{g}\left(r\right)$. Since each point $r^{\mu }\equiv r^{\mu }\left(s\right)$ can be crossed by infinite arbitrary geodetics having different tangent 4-vectors $t^{\mu }\equiv t^{\mu }\left(s\right)$ it follows that the wave-function parameterization ψ≡ψ(g,r,s) may generally depend explicitly on the choice of the geodetics, i.e., on $t^{\mu }$ too. In particular, ψ(g,r,s) will be assumed to contain the following smooth dependences:

(1) *Explicit g-dependence:* ψ(g,r,s) is assumed to be a $C^{\left(2\right)}$, smoothly differentiable complex function of the continuum Lagrangian variables $g=\left\{g_{\mu \nu }\right\}$.

(2) *Explicit and implicit s-dependences:* ψ(g,r,s) may depend both explicitly and implicitly on *s*. The implicit dependence occurs via r(s) and t(s) and therefore also in terms of the prescribed metric tensor through its explicit spatial dependence $\hat{g}\left(r\right)$. These *s*-dependences will all be assumed to realize in terms of ψ(g,r,s) a $C^{\left(1\right)}$, smoothly differentiable function of *s*.

The next step is the identification of the quantum-wave equation which determines the CQG state ψ(g,r,s). This task is achieved by means of the *CQG-wave equation* [10]. Written again in the Eulerian form, the latter is realized by the initial-value problem

(16) $$\left\{\begin{array}{c} i\hbar \frac{\partial }{\partial s}\psi \left(g,r,s\right)=\left[H_{R},\psi \left(g,r,s\right)\right]\equiv H_{R}\psi \left(g,r,s\right),\\ \psi \left(g,r\left(s_{o}\right)=r_{o},s_{o}\right)=\psi_{o}\left(g,r_{o}\right), \end{array}\right.$$

where in the first equation the squared-brackets denote the quantum commutator in standard notation, while the operator $\frac{\partial }{\partial s}$ appearing in the scalar Equation (16) in the Eulerian representation coincides with $\frac{\partial }{\partial s}\equiv \frac{d}{ds},$ being $\frac{d}{ds}$ the total covariant *s*-derivative in Equation (14) again prescribed in terms of the background metric tensor $\hat{g}\left(r\right)$. Notice that the initial-value problem in Equation (16) can be represented equivalently in terms of the initial quantum fluid fields

(17) $$\left\{\begin{array}{c} \rho \left(g,r\left(s_{o}\right)=r_{o},s_{o}\right)=\rho_{o}\left(g,r_{o}\right),\\ S^{\left(q\right)}\left(g,r\left(s_{o}\right)=r_{o},s_{o}\right)=S_{o}^{\left(q\right)}\left(g,r_{o}\right). \end{array}\right.$$

As such, provided ψ(g,r,s) is suitably smooth the solution of Equation (16) is unique. Thus, Equation (16) realizes a hyperbolic evolution equation, i.e., a first-order PDE with respect to the proper time *s*. In the same equation $H_{R}$ denotes the quantum Hamiltonian operator characteristic of CQG-theory, to be expressed in terms of the relevant quantum momentum operator, namely $\pi_{\mu \nu }^{\left(q\right)}=-\frac{i\hbar }{\alpha L}\frac{\partial }{\partial g^{\mu \nu }}$. Here, the partial derivative is performed keeping constant all remaining variables appearing in ψ(g,r,s), while *L* and α are, respectively, a suitably-defined 4-scalar scale-length and a dimensional 4-scalar parameter related to the universal constant $\kappa =\frac{c^{3}}{16\pi G}$ (see again [10]). Then, the quantum Hamiltonian operator $H_{R}$ takes the form

(18) $$H_{R}\equiv T_{R}^{\left(q\right)}+V\left(g,r,s\right),$$

with $T_{R}^{\left(q\right)}\left(x,\hat{g}\right)\equiv \frac{1}{2\alpha L}\pi_{\mu \nu }^{\left(q\right)}\pi^{(q)\mu \nu }$ and V(g,r,s) being, respectively, the effective kinetic energy operator and the effective potential energy

(19) $$\left\{\begin{array}{c} V\left(g,r,s\right)\equiv \sigma V_{o}\left(g\right)+\sigma V_{F}\left(g,r,s\right),\\ V_{o}\left(g\right)\equiv \alpha Lh\left[g^{\mu \nu }{\hat{R}}_{\mu \nu }-2\Lambda \right],\\ V_{F}\equiv \frac{\alpha L}{k}hL_{F}\left(g,r,s\right), \end{array}\right.$$

with $V_{o}\left(g\right)$ and $V_{F}\left(g,r,s\right)$ identifying the vacuum and external effective contributions to the effective potential V(g,r,s). Here, the notation is given according to [10]. Thus, all hatted quantities are evaluated with respect to the background metric tensor $\hat{g}$ only while the multiplicative 4-scalar gauge function σ is taken to be σ=−1. In addition, V≡V(g,r,s) itself is determined up to an arbitrary additive gauge transformation of the form $V\to V^{\prime }=V-{\left.\frac{d}{ds}F\left(g,r\left(s\right)s\right)\right|}_{g}$, being F(g,r(s)s) a 4-scalar function of the form

(20) $$F\left(g,r\left(s\right)s\right)=g_{\mu \nu }G^{\mu \nu }\left(\hat{g},r\left(s\right),s\right)+F_{1}\left(\hat{g},r\left(s\right),s\right),$$

with $G^{\mu \nu }\left(\hat{g},r\left(s\right),s\right)$ and $F_{1}\left(\hat{g},r\left(s\right),s\right)$ denoting, respectively, a 4-tensor and a 4-scalar smoothly differential real gauge, i.e., arbitrary, functions. A characteristic element of CQG-theory is the quantity h≡h(g) first introduced in [7].The prescription of h(g) is obtained in terms of a polynomial function of $g\equiv \hat{g}+\delta g$, with δg being an in principle arbitrary variational displacement so that according to the same reference (see also [7]):

(21) $$h\left(g\right)=2-\frac{1}{4}\left({\hat{g}}^{\alpha \beta }+\delta g^{\alpha \beta }\right)\left({\hat{g}}^{\mu \nu }+\delta g^{\mu \nu }\right){\hat{g}}_{\alpha \mu }{\hat{g}}_{\beta \nu }.$$

As a final remark, we notice that the Eulerian CQG-state defined by the complex function ψ(g,r,s) can always be cast in the form of an exponential representation via the Madelung representation recalled above. Elementary algebra [10,11] then shows that, based on the quantum-wave Equation (16), the same quantum fluid fields necessarily fulfill the corresponding set of Eulerian CQG-quantum hydrodynamic equations. In the Eulerian representation, upon identifying again $\frac{d}{ds}$ with the total covariant s-derivative operator in Equation (14), these are realized respectively by the continuity and quantum Hamilton-Jacobi equations:

(22) $$\left\{\begin{array}{c} \frac{d\rho (g,r,s)}{ds}+\frac{\partial }{\partial g_{\mu \nu }}\left(\rho \left(g,r,s\right)V_{\mu \nu }\left(g,r,s\right)\right)=0,\\ \frac{dS^{\left(q\right)}\left(g,r,s\right)}{ds}+H_{c}\left(g,r,s\right)=0, \end{array}\right.$$

which represent a set of evolution PDEs for the quantum fluid fields ρ(g,r,s) and $S^{\left(q\right)}\left(g,r,s\right)$. Notice that, in the previous equations, $V_{\mu \nu }\left(q,s\right)$ and $H_{c}\left(g,r,s\right)$ denote, respectively, the tensor “velocity” field $V_{\mu \nu }\left(g,r,s\right)=\frac{1}{\alpha L}\frac{\partial S^{\left(q\right)}\left(g,r,s\right)}{\partial g^{\mu \nu }}$ and the effective quantum Hamiltonian density

(23) $$H_{c}\left(g,r,s\right)=\frac{1}{2\alpha L}\frac{\partial S^{\left(q\right)}\left(g,r,s\right)}{\partial g^{\mu \nu }}\frac{\partial S^{\left(q\right)}\left(g,r,s\right)}{\partial g_{\mu \nu }}+V_{QM}\left(g,r,s\right)+V\left(g,r,s\right),$$

with

(24) $$T=\frac{1}{2\alpha L}\frac{\partial S^{\left(q\right)}\left(g,r,s\right)}{\partial g^{\mu \nu }}\frac{\partial S^{\left(q\right)}\left(g,r,s\right)}{\partial g_{\mu \nu }}$$

being the effective kinetic energy. In addition, V(g,r,s) and $V_{QM}\left(g,r,s\right)$ identify, respectively, the effective potential density (19) and the Bohm effective quantum potential

(25) $$V_{QM}\left(g,r,s\right)\equiv -\frac{\hbar^{2}}{8\alpha L}\frac{\partial \ln\rho (g,r,s)}{\partial g^{\mu \nu }}\frac{\partial \ln\rho (g,r,s)}{\partial g_{\mu \nu }}-\frac{\hbar^{2}}{4\alpha L}\frac{\partial^{2}\ln\rho \left(g,r,s\right)}{\partial g_{\mu \nu }\partial g^{\mu \nu }},$$

or equivalently $V_{QM}\left(g,r,s\right)\equiv \frac{\hbar^{2}}{8\alpha L}\frac{\partial \ln\rho (g,r,s)}{\partial g^{\mu \nu }}\frac{\partial \ln\rho (g,r,s)}{\partial g_{\mu \nu }}-\frac{\hbar^{2}}{4\alpha L}\frac{\partial^{2}\rho \left(g,r,s\right)}{\rho \partial g_{\mu \nu }\partial g^{\mu \nu }}.$

## 4. Lagrangian Path (Bohmian) Representation

It is well known that in the non-relativistic framework the Bohmian interpretation of quantum mechanics provides the corresponding trajectory-based Lagrangian Path representation (*LP-representation*) of the Schroedinger quantum-wave equation (see [73,74] for a review of the topic). The intrinsic similarity with the CQG-wave equation suggests that an analogous Lagrangian representation is possible also for the same equation, so that as a consequence, a “Bohmian” trajectory-based interpretation can be achieved in the context of CQG-theory too. In both cases, in fact, the Lagrangian representation is based on the introduction of a suitable family of configuration-space trajectories, or Lagrangian Paths (LP), which for each “point” of the appropriate quantum configuration space are unique. In the context of CQG-theory, the LP-representation involves the introduction for all s∈I of the correspondence (8), with $\delta g_{\mu \nu }\equiv \delta g_{L\mu \nu }\left(s\right)\in U_{g}$ belonging to a suitable curve $\left\{g_{L}\left(s\right),\forall s\in I\right\}$ of the configuration space $U_{g}$ denoted as *Lagrangian path*. Consequently, each LP is identified with a well-defined characteristics associated with the tensor velocity field $V_{\mu \nu }\left(g,r,s\right)$. For definiteness, based on the tensor decomposition (5), the LP-representation involves parameterizing all quantum fields, and in particular the quantum state, in terms of $g_{L\mu \nu }\left(s\right)$ thus letting $\psi \equiv \psi (g_{L\mu \nu }\left(s\right),r\left(s\right),s)$. As such, $\delta g_{L\mu \nu }\left(s\right)$ is constructed in such a way that its “tangent” coincides with the local value of the tensor velocity field $V_{\mu \nu }$, namely so that they fulfill the initial-value problem

(26) $$\left\{\begin{array}{c} \frac{D}{Ds}g_{L\mu \nu }\left(s\right)=V_{\mu \nu }\left(g_{L}\left(s\right),s\right),\\ g_{\mu \nu }\left(s_{o}\right)=g_{\mu \nu }^{\left(o\right)}. \end{array}\right.$$

Here, $\frac{D}{Ds}$ identifies the LP-derivative (or covariant *s*-derivative) realized by the operator

(27) $$\frac{D}{Ds}\equiv {\left.\frac{d}{ds}\right|}_{\delta g_{L\mu \nu }\left(s\right)}+V_{\mu \nu }\left(g_{L}\left(s\right),s\right)\frac{\partial }{\partial \delta g_{L\mu \nu }},$$

where the two terms on the R.H.S. of Equation (27) identify respectively the covariant *s*-derivative performed at constant $\delta g_{L\mu \nu }\equiv \delta g_{L\mu \nu }\left(s\right)$, namely

(28) $${\left.\frac{d}{ds}\right|}_{\delta g_{L\mu \nu }\left(s\right)}\equiv {\left.\frac{D}{Ds}\right|}_{\delta g_{\mu \nu }}={\left[{\left.\frac{\partial }{\partial s}\right|}_{r}+t^{\alpha }\nabla_{\alpha }\right]}_{\delta g_{L\mu \nu }},$$

and the convective derivative performed with respect to the Lagrangian coordinates $\delta g_{L\mu \nu }\left(s\right)$ while keeping constant $\hat{g}\left(r\right)\equiv \left\{{\hat{g}}_{\mu \nu }\left(r\left(s\right)\right)\right\}$. In view of Equation (5), Equation (26) can be written as

(29) $$\left\{\begin{array}{c} \frac{D}{Ds}\delta g_{L\mu \nu }\left(s\right)=V_{\mu \nu }\left(\hat{g}\left(r\right)+\delta g_{L}\left(s\right),s\right),\\ \delta g_{L\mu \nu }\left(s_{o}\right)=\delta g_{\mu \nu }^{\left(o\right)}. \end{array}\right.$$

Consequently, Equation (29) can be integrated to give

(30) $$\delta g_{L\mu \nu }\left(s\right)=\delta g_{\mu \nu }^{\left(o\right)}+\int_{s_{o}}^{s}ds^{\prime }V_{\mu \nu }\left(\hat{g}\left(r\right)+\delta g_{L}\left(s^{\prime }\right),s^{\prime }\right),$$

which determines the LP itself, namely the trajectory $\left\{g_{L}\left(s\right),\forall s\in I\right\}\equiv \left\{g_{L}\left(s\right)\equiv \hat{g}\left(r\right)+\delta g_{L}\left(s\right),\forall s\in I\right\}$.However, if $H_{\mu \nu }\equiv H_{\mu \nu }\left(\hat{g}\left(r\right)\right)$ denotes an arbitrary smoothly-differentiable tensor function of $\hat{g}\left(r\right)$, it is obvious that also the arbitrary additive tensor quantity of the form $\frac{D}{Ds}\left[\delta g_{L\mu \nu }\left(s\right)+H_{\mu \nu }\left(\hat{g}\left(r\right)\right)\right]$ satisfies identically Equation (29). Since uniqueness of the solution $\delta g_{L\mu \nu }\left(s\right)$ given by Equation (30) is warranted by prescribing $\delta g_{\mu \nu }^{\left(o\right)},$ the mapping

(31) $$g_{L\mu \nu }\left(s_{o}\right)=g_{\mu \nu }^{\left(o\right)}\Leftrightarrow g_{L\mu \nu }\left(s\right)$$

identifies a classical dynamical system (CDS), i.e., a diffeomeorphism mutually mapping in each other two arbitrary points $g_{L\mu \nu }\left(s_{o}\right)$ and $g_{L\mu \nu }\left(s\right)$ which belong to the same LP. Consequently, the Liouville theorem warrants that the Jacobian determinant of the transformation (31) is

(32) $$\left|\frac{\partial \delta g_{L}\left(s\right)}{\partial \delta g_{L}\left(s_{o}\right)}\right|=\exp\left\{\int_{s_{o}}^{s}ds^{\prime }\frac{\partial V_{\mu \nu }\left(g_{L}\left(s^{\prime }\right),s^{\prime }\right)}{\partial g_{L\mu \nu }\left(s^{\prime }\right)}\right\}.$$

The Lagrangian representation of CQG-theory is then achieved by means of the formal replacement $g\to g_{L}\left(s\right)$ to be made in the quantum wave-function, i.e., introducing in the CQG-wave Equation (16) the LP-parameterization $\psi =\psi (g_{L}\left(s\right),s)$ and similarly for the quantum fluid fields, namely

(33) $$\left\{\rho ,S^{\left(q\right)}\right\}\equiv \left\{\rho \left(g_{L}\left(s\right),s\right),S^{\left(q\right)}\left(g_{L}\left(s\right),s\right)\right\}.$$

As a result, in terms of the tensor velocity field in the LP-representation, namely $V_{\mu \nu }\left(g_{L}\left(s\right),s\right)\equiv \frac{1}{\alpha L}\frac{\partial S^{\left(q\right)}\left(g_{L}\left(s\right),r\left(s\right),s\right)}{\partial \delta g_{L}^{\mu \nu }\left(s\right)}$, the quantum hydrodynamic Equation (22) can be set at once in the corresponding Lagrangian form. To obtain them one notices preliminarily that

(34) $$\frac{D}{Ds}S^{\left(q\right)}\left(g_{L}\left(s\right),s\right)\equiv \frac{d}{ds}S^{\left(q\right)}\left(g_{L}\left(s\right),s\right)+V_{\mu \nu }\left(g_{L}\left(s\right),s\right)\frac{\partial S^{\left(q\right)}\left(g_{L}\left(s\right),s\right)}{\partial g_{L\mu \nu }\left(s\right)},$$

with $\frac{D}{Ds}$ and $\frac{d}{ds}$ identifying respectively the LP-derivative in Equation (27) and the total covariant *s*-derivative operator in Equation (14). Consequently, the LP-representation of the quantum fluid Equation (22) is given by the PDEs

(35) $$\left\{\begin{array}{c} \frac{D}{Ds}\rho \left(g_{L}\left(s\right),s\right)=-\rho \left(g_{L}\left(s\right),s\right)\frac{\partial V_{\mu \nu }\left(g_{L}\left(s\right),s\right)}{\partial g_{L\mu \nu }\left(s\right)},\\ \frac{D}{Ds}S^{\left(q\right)}\left(g_{L}\left(s\right),s\right)=V_{\mu \nu }\left(g_{L}\left(s\right),s\right)\frac{\partial S^{\left(q\right)}\left(g_{L}\left(s\right),s\right)}{\partial g_{L\mu \nu }\left(s\right)}-H_{c}\left(g_{L}\left(s\right),s\right), \end{array}\right.$$

where $H_{c}\left(g_{L}\left(s\right),s\right)$ identifies the effective quantum Hamiltonian density in Equation (23) parameterized in terms of $g_{L}\left(s\right)$. Thus, in particular, the continuity equation (first part of Equation (35)) can be formally integrated to give the LP-parameterized integral continuity equation

(36) $$\rho \left(g_{L}\left(s\right),s\right)=\rho \left(g_{L}\left(s_{o}\right),s_{o}\right)\exp\left\{-\int_{s_{o}}^{s}ds^{\prime }\frac{\partial V_{\mu \nu }\left(g_{L}\left(s^{\prime }\right),s^{\prime }\right)}{\partial g_{L\mu \nu }\left(s^{\prime }\right)}\right\},$$

with $\rho \left(g_{L}\left(s_{o}\right),s_{o}\right)\equiv \rho \left(g_{L}\left(s_{o}\right),r\left(s_{o}\right)=r_{o},s_{o}\right)$ denoting the initial quantum PDF, namely

(37) $$\rho \left(g_{L}\left(s_{o}\right),r\left(s_{o}\right)=r_{o},s_{o}\right)=\rho_{o}\left(g_{L}\left(s_{o}\right),r_{o}\right).$$

Together with Liouville theorem in Equation (32) this implies therefore the conservation laws

(38) $$d\left(g_{L}\left(s\right)\right)\rho \left(g_{L}\left(s\right),s\right)=d\left(g_{L}\left(s_{o}\right)\right)\rho \left(g_{L}\left(s_{o}\right),s_{o}\right),$$

(39) $$\int_{U_{g}}d\left(g_{L}\left(s\right)\right)\rho \left(g_{L}\left(s\right),s\right)=\int_{U_{g}}d\left(g_{L}\left(s_{o}\right)\right)\rho \left(g_{L}\left(s_{o}\right),s_{o}\right)=1,$$

which warrant, consistent with the quantum unitarity principle, the conservation of the quantum probability in $U_{g}$.

We conclude this section noting that from a mathematical viewpoint the Lagrangian formulation of CQG-theory is actually realized solely by the LP-parameterized quantum hydrodynamic Equations (35). Therefore, the Lagrangian and Eulerian quantum hydrodynamic equations are manifestly equivalent. This suggests that a Bohmian interpretation of the Lagrangian-path representation of the CQG-theory is in principle possible. However, just as in the case of the Schroedinger equation (see related discussion in [22]), a basic difficulty of such an interpretations lies in the uniqueness feature, and consequently the intrinsic deterministic character, of each LP. Such a property, in fact, appears potentially in contradiction with the notion of quantum measurement holding in the context of CQG-theory and the validity of Heisenberg inequalities [11].

## 5. Generalized Lagrangian Path Representation

The considerations indicated above lead us to introduce the notion of Generalized Lagrangian Path (GLP) and of the corresponding GLP-representation obtained in this way for the quantum wave-function and quantum fluids fields. As anticipated above (see Introduction) this is achieved by means of the introduction of a suitable set of intrinsically non-unique and stochastic trajectories, to be referred to as generalized Lagrangian paths (GLPs), in terms of which the quantum wave-equation, as well as the corresponding set of quantum fluid fields and quantum hydrodynamic equations, can be parameterized. In the context of CQG-theory the mathematical problem of formulating its GLP-representation involves the introduction for all s∈I of a suitable correspondence of the type

(40) $$s\to \delta G_{L}\left(s\right),$$

referred to as *GLP-map*. Then, upon invoking the tensor decomposition (Equation (9)), a GLP is the curve $\left\{G_{L}\left(s\right),\forall s\in I\right\}$ of the quantum configuration space $U_{g}$ which is defined by Equation (10) and is realized by the ensemble of ”points” of $U_{g}$ spanned by the tensor field $G_{L}\left(s\right)\equiv G\left(s\right)$ and obtained varying s∈I. The underlying basic idea is therefore to replace a single LP, prescribed in terms of a solution of the initial-value problem in Equation (26), with an infinite set of stochastic trajectories, each one identified with a single GLP and characterized by a unique choice of a suitable stochastic tensor $\Delta g=\left\{\Delta g_{\mu \nu }\right\}$. This effectively involves introducing a parameter-dependent mapping of the type

(41) $$\left\{g_{L}\left(s\right),\forall s\in I\right\}\to \left\{G_{L}\left(s\right),\forall s\in I\right\},$$

whose realization depends on the prescription of $\Delta g=\left\{\Delta g_{\mu \nu }\right\}$. Then, the GLP-map in Equation (41) is realized by means of the following two requirements.

- *GLP Requirement #1*- The first one is realized by prescribing $\delta G_{L\mu \nu }\left(s\right)$ in terms of the displacement tensor $\delta g_{L\mu \nu }\left(s\right)$ which is determined according to Equation (5). This yields therefore the identity
  (42) $$G_{L\mu \nu }\left(s\right)={\hat{g}}_{\mu \nu }\left(r\right)+\delta g_{L\mu \nu }\left(s\right)-\Delta g_{\mu \nu },$$
  with Δg denoting the *stochastic displacement* 4-*tensor*
  (43) $$\Delta g=g-G_{L}\left(s\right)\equiv \delta g-\delta G_{L}\left(s\right).$$
  Notice that, here, $g_{\mu \nu }=g_{L\mu \nu }\left(s\right)$, and hence $\delta g_{\mu \nu }\equiv \delta g_{L\mu \nu }\left(s\right)$. Consequently, it is understood that Δg must be endowed with a suitable stochastic PDF to be suitably prescribed. In this regards, taking Δg as an independent stochastic variable, it is natural to assume that the same PDF should be a stationary and spatially uniform probability distribution, i.e., a function independent of r, *s* as well as $\delta g_{L}\left(s\right)$, but still allowed to depend in principle on the prescribed metric tensor ${\hat{g}}_{\mu \nu }\left(r\right)$. More precisely, this means assuming the same PDF to be realized in terms of a smoothly differentiable and strictly positive function of the form
  (44) $$f=f\left(\Delta g,\hat{g}\right).$$
  Hence, the corresponding notion of stochastic average for an arbitrary smooth function X(Δg,r,s) is prescribed in terms of the weighted integral
  (45) $${\langle X(\Delta g,r,s)\rangle }_{stoch}\equiv \int_{U_{g}}d\left(\Delta g\right)X\left(\Delta g,r,s\right)f\left(\Delta g,\hat{g}\right),$$
  to be performed on the configuration space $U_{g}$. In particular, besides the prescription (44), $f\left(\Delta g,\hat{g}\right)$ should be prescribed so that the following stochastic averages are also fulfilled:
  (46) $$\left\{\begin{array}{c} {\langle 1\rangle }_{stoch}\equiv \int_{U_{g}}d\left(\Delta g\right)f\left(\Delta g,\hat{g}\right)=1,\\ {\langle \Delta g_{\mu \nu }\rangle }_{stoch}\equiv \int_{U_{g}}d\left(\Delta g\right)\Delta g_{\mu \nu }f\left(\Delta g,\hat{g}\right))=\pm {\hat{g}}_{\mu \nu }\left(r\right),\\ \sigma_{\Delta g}^{2}\equiv {\langle {\left(\Delta g-{\langle \Delta g\rangle }_{stoch}\right)}^{2}\rangle }_{stoch}\equiv \\ \int_{U_{g}}d\left(\Delta g\right){\left(\Delta g-{\langle \Delta g\rangle }_{stoch}\right)}^{2}f\left(\Delta g,\hat{g}\right)=r_{th}^{2}, \end{array}\right.$$
  with ${\left(\Delta g-{\langle \Delta g\rangle }_{stoch}\right)}^{2}\equiv \left[\Delta g_{\mu \nu }-{\langle \Delta g_{\mu \nu }\rangle }_{stoch}\right]\left[\Delta g^{\mu \nu }-{\langle \Delta g^{\mu \nu }\rangle }_{stoch}\right]$ and $\sigma_{\Delta g}$ denoting the standard deviation of Δg to be identified with the dimensionless 4-scalar parameter $r_{th}^{2}>0$. Notice in addition that, here, for consistency with the same assumption in Equation (44), $r_{th}^{2}$ must be assumed to be a non-vanishing constant, i.e., independent of both (r,s).
- *GLP Requirement #2*- The second one is obtained requiring that $\Delta g=\left\{\Delta g_{\mu \nu }\right\}$ is constant for all s∈I and for an arbitrary Lagrangian Path, i.e., it is prescribed so that identically for all $s,s_{o}\in I$ it occurs that
  (47) $$\Delta g_{\mu \nu }\left(s\right)=\Delta g_{\mu \nu }\left(s_{o}\right).$$
  Notice that here $\frac{D}{Ds}\delta g_{L\mu \nu }\left(s\right)=\frac{D}{Ds}\delta G_{L\mu \nu }\left(s\right)\equiv V_{\mu \nu }\left(G_{L}\left(s\right),\Delta g,s\right)),$ with $V_{\mu \nu }\left(G_{L}\left(s\right),\Delta g,s\right)$ being the tensor velocity field in the GLP-representation, namely
  (48) $$V_{\mu \nu }\left(G_{L}\left(s\right),\Delta g,s\right)=\frac{1}{\alpha L}\frac{\partial S^{\left(q\right)}\left(G_{L}\left(s\right),\Delta g,s\right)}{\partial \delta g_{L}^{\mu \nu }\left(s\right)},$$
  while $\frac{D}{Ds}$ is the Lagrangian derivative defined above (see Equation (27)). As a result, the constraint condition (47) necessarily implies also that
  (49) $$\frac{D}{Ds}\Delta g_{\mu \nu }\equiv \frac{D}{Ds}\delta g_{L\mu \nu }\left(s\right)-\frac{D}{Ds}\delta G_{L\mu \nu }\left(s\right)\equiv 0.$$

As a consequence of Requirements #1 and #2, for all s∈I, the correspondence in Equation (40) is uniquely established, in the sense that, for each determination of the stochastic displacement Δg, $G_{L}\left(s,\Delta g\right)\equiv G_{L}\left(s\right)$ belongs to a uniquely-prescribed curve $\left\{G_{L}\left(s\right),\forall s\in I\right\},$ identifying a GLP which spans the quantum configuration space $U_{g}$. More precisely, a generic GLP $\left\{G_{L}\left(s\right),\forall s\in I\right\}$ is identified with the integral curve determined by the GLP-initial-value problem

(50) $$\left\{\begin{array}{c} \frac{D}{Ds}\delta G_{L\mu \nu }\left(s\right)=V_{\mu \nu }\left(G_{L}\left(s\right),\Delta g,s\right),\\ \delta G_{L\mu \nu }\left(s_{o}\right)=\delta g_{\mu \nu }^{\left(o\right)}-\Delta g_{\mu \nu }. \end{array}\right.$$

In addition, here, the map $G_{L}\left(s_{o}\right)\Leftrightarrow G_{L}\left(s\right)$ defines again a classical dynamical system with Jacobian determinant

(51) $$\left|\frac{\partial G_{L}\left(s\right)}{\partial G_{L}\left(s_{o}\right)}\right|=\exp\left\{\int_{s_{o}}^{s}ds^{\prime }\frac{\partial V_{\mu \nu }\left(G_{L}\left(s^{\prime }\right)+\Delta g,s^{\prime }\right)}{\partial g_{L\mu \nu }\left(s^{\prime }\right)}\right\}.$$

The ensemble of integral curves $\left\{G_{L}\left(s\right),\forall s\in I\right\}$ obtained by varying Δg in $U_{g}$ identifies therefore an infinite set of GLP which are associated with the tensor velocity field $V_{\mu \nu }\left(G_{L}\left(s\right)+\Delta g,s\right)$. One notices, however, that by construction,

(52) $$V_{\mu \nu }\left(G_{L}\left(s\right)+\Delta g,s\right)=V_{\mu \nu }\left(g_{L}\left(s\right),s\right).$$

Thus, the same infinite set of GLP is actually associated with the same local value of the tensor velocity field $V_{\mu \nu }\left(g_{L}\left(s\right),s\right)$. Thus, in contrast with the LP defined above (in terms of Equation (26)), this means that the GLP which are associated with the local tensor velocity field $V_{\mu \nu }\left(g_{L}\left(s\right),s\right)$ are non-unique (and actually infinite), each one being determined by Δg. Precisely because the same trajectories are stochastic and hence non-unique, such a feature is in principle compatible with the possible interpretation of the GLPs as *physical quantum trajectories in the configurations space* $U_{g}$. Nevertheless, the prerequisite for making actually possible such an interpretation is, ultimately, the ontological equivalence of the GLP-parameterization for the quantum state ψ with the “standard” Eulerian representation of the same quantum wave-function. In other words, the adoption of the GLP and in particular the prescription of the stochastic PDF f(Δg) associated with the same constant stochastic displacement tensor Δg (see Equation (44)), should be possible leaving unchanged the axioms of CQG-theory.

For definiteness, let us now pose the problem of introducing explicitly the parameterization of the quantum fluid fields and the related GLP-representation of the QHE. In principle, this can simply be obtained from the corresponding LP-parameterization indicated above noting that δg(s)=Δg+δG(s). However, in formal analogy with the GLP-approach to non-relativistic quantum mechanics earlier indicated, a more general parameterization in terms of the stochastic displacement tensor field Δg, to be referred in the sequel as GLP-parameterization, is possible. This involves assuming that the CQG-wave function may be of the type

(53) $$\psi =\psi (G_{L}\left(s\right),\Delta g,s),$$

i.e., to include also an explicit dependence in terms of $\Delta g\equiv \left\{\Delta g_{\mu \nu }\right\}$. Therefore, the corresponding GLP-parameterization of the quantum fluid fields is taken of the form

(54) $${\left\{\rho ,S^{\left(q\right)}\right\}}_{\left(s\right)}\equiv \left\{\rho \left(G_{L}\left(s\right),\Delta g,s\right),S^{\left(q\right)}\left(G_{L}\left(s\right),\Delta g,s\right)\right\}.$$

Nevertheless, the quantum hydrodynamic Equation (22), when expressed in the GLP-parameterization, remain formally analogous to those obtained in the LP-parameterization (see Equation (35)), so that the same equations must determine the map

(55) $${\left\{\rho ,S^{\left(q\right)}\right\}}_{\left(s_{o}\right)}\equiv \left\{\rho_{o},S_{o}^{\left(q\right)}\right\}\to {\left\{\rho ,S^{\left(q\right)}\right\}}_{\left(s\right)},$$

with $\left\{\rho_{o},S_{o}^{\left(q\right)}\right\}$ being suitable initial quantum fluid fields. Hence, for consistency, these should be again assumed of the form

(56) $$\left\{\rho_{o},S_{o}^{\left(q\right)}\right\}\equiv \left\{\rho_{o}\left(G_{L}\left(s_{o}\right),\Delta g\right),S_{o}^{\left(q\right)}\left(G_{L}\left(s_{o}\right),\Delta g\right)\right\}.$$

In detail, in the GLP-representation the quantum hydrodynamic Equation (35) are now realized by the PDEs

(57) $$\left\{\begin{array}{c} \frac{D}{Ds}\rho \left(G_{L}\left(s\right),\Delta g,s\right)=-\rho \left(G_{L}\left(s\right),\Delta g,s\right)\frac{\partial V_{\mu \nu }\left(G_{L}\left(s\right),\Delta g,s\right)}{\partial g_{L\mu \nu }\left(s\right)},\\ \frac{D}{Ds}S^{\left(q\right)}\left(G_{L}\left(s\right),\Delta g,s\right)=K_{c}\left(G_{L}\left(s\right),\Delta g,s\right), \end{array}\right.$$

representing, respectively, the GLP-parameterized quantum continuity and H-J equations, where

(58) $$K_{c}\left(G_{L}\left(s\right),\Delta g,s\right)=V_{\mu \nu }\left(G_{L}\left(s\right),\Delta g,s\right)\frac{\partial S^{\left(q\right)}\left(G_{L}\left(s,\Delta g\right),\Delta g,s\right)}{\partial g_{L\mu \nu }\left(s\right)}-H_{c}\left(G_{L}\left(s\right),\Delta g,s\right)$$

and $H_{c}\left(G_{L}\left(s\right),\Delta g,s\right)$ identifies now the effective quantum Hamiltonian density in Equation (23) expressed in terms of the GLP-parameterization. Thus, from Equation (23), it follows that

(59) $$H_{c}\left(G_{L}\left(s\right),\Delta g,s\right)=T\left(G_{L}\left(s\right),\Delta g,s\right)-V\left(G_{L}\left(s\right),\Delta g,s\right)-V_{QM}\left(G_{L}\left(s\right),\Delta g,s\right),$$

with $T\equiv T(G_{L}\left(s\right),\Delta g,s)$, $V\equiv V(G_{L}\left(s\right),\Delta g,s)$ and $V_{QM}\equiv V_{QM}\left(G_{L}\left(s\right),\Delta g,s\right)$ denoting now in terms of the GLP-parameterization respectively the effective kinetic energy and classical potential density given by Equations (24), (19) and the Bohm effective quantum potential in Equation (25). Thus, regarding the representation of the effective potential energy V, and in particular its vacuum contribution $V_{o}\equiv V_{o}\left(G_{L}\left(s\right),\Delta g,s\right)$ (see Equation (19)), to be used in the context of the GLP-approach, one notices that the displacement 4-tensor δg entering the expression of the variational parameter in Equation (21) remains non-unique. One notices that, due to its arbitrariness, the displacement 4-tensor can always be identified with δg≡Δg, being Δg the stochastic constant displacement field tensor introduced above (see Equation (11)), so that actually h(g) can be conveniently represented as

(60) $$h\left(\hat{g}+\Delta g\right)=2-\frac{1}{4}\left({\hat{g}}^{\alpha \beta }+\Delta g^{\alpha \beta }\right)\left({\hat{g}}^{\mu \nu }+\Delta g^{\mu \nu }\right){\hat{g}}_{\alpha \mu }{\hat{g}}_{\beta \nu },$$

while the vacuum effective potential becomes:

(61) $$V_{o}\left(G_{L}\left(s\right),\Delta g,s\right)\equiv \sigma \alpha Lh\left(\hat{g}+\Delta g\right)\left[\left({\hat{g}}_{pq}\left(s\right)+\Delta g_{pq}\right){\hat{g}}^{pq}\left(r\right)-2\right]\Lambda .$$

Useful implications of the GLP-representation in Equations (53)–(54) follow by inspection of the GLP-quantum continuity equation (see first equation in Equation (57)) obtained above. The first one follows by noting that the same equation implies also

(62) $$\frac{D}{Ds}\ln\rho \left(G_{L}\left(s\right),\Delta g,s\right)=-\frac{\partial V_{\mu \nu }\left(G_{L}\left(s\right),\Delta g,s\right)}{\partial g_{L\mu \nu }\left(s\right)},$$

so that its formal integration generates the map $\rho \left(G_{L}\left(s_{o}\right),\Delta g,s_{o}\right)\to \rho \left(G_{L}\left(s\right),\Delta g,s\right),$ with $\rho (G_{L}\left(s\right),\Delta g,s)$ denoting the proper-time evolved quantum PDF, namely

(63) $$\rho \left(G_{L}\left(s\right),\Delta g,s\right)=\rho \left(G_{L}\left(s_{o}\right),\Delta g,s_{o}\right)\exp\left\{-\int_{s_{o}}^{s}ds^{\prime }\frac{\partial V_{\mu \nu }\left(G_{L}\left(s^{\prime }\right),\Delta g,s^{\prime }\right)}{\partial g_{L\mu \nu }\left(s^{\prime }\right)}\right\}.$$

Notice that the integration on the R.H.S is performed along the GLP-trajectory $\left\{G_{L}\left(s,\Delta g\right),\forall s\in I\right\}$, i.e., for a prescribed constant stochastic displacement 4-tensor Δg, while $\rho (G_{L}\left(s_{o}\right),\Delta g,s_{o})$ identifies the initial, and in principle still arbitrary, PDF. The second implication concerns the quantum H-J equation itself. In fact, the formal solution in Equation (63) permits to cast it in terms of an (implicit) equation for the GLP-parameterized quantum phase-function $S^{\left(q\right)}\left(G_{L}\left(s\right),\Delta g,s\right)$ only. Consequently, provided an explicit realization is reached for the GLP-trajectory $\left\{G_{L}\left(s\right),\forall s\in I\right\}$, by solving the initial-value problem (50), the same H-J equation should uniquely determine the corresponding solution $S^{\left(q\right)}\left(G_{L}\left(s\right),\Delta g,s\right)$ as a real function of Δg and *s* only. A notable feature worth to be stressed here is about the prescription of the same initial PDF $\rho (G_{L}\left(s_{o}\right),\Delta g,s_{o})$. This manifestly generally differs from the one considered above in the case of the LP-parameterization (see Equation (37)), where no explicit Δg−dependences was assumed. In fact, consistent with the GLP-parameterization introduced above (see Equation (54)), this is now taken of the form (56). This means that it may include in particular an admissible choice for the initial PDF provided by a probability density of the form

(64) $$\rho \left(G_{L}\left(s_{o}\right),\Delta g,s_{o}\right)=\rho_{o}\left(\Delta g+\hat{g}\left(r_{o}\right)\right),$$

with $\rho (\Delta g+\hat{g}\left(r_{o}\right))$ to be determined as indicated below.

## 6. GLP Approach: Determination of the Stochastic PDF for Δg and of the Quantum PDF

The problem addressed in this section is twofold. First, it concerns the identification of the stochastic probability density $f(\Delta g,{\hat{g}}_{\mu \nu })$ which is associated with the stochastic displacement tensor field $\Delta g\equiv \left\{\Delta g_{\mu \nu }\right\}$ and is consistent with the requirements indicated above, i.e., Equation (44), together with the aforementioned constraint conditions in Equation (46). Second, it deals with the prescription of the CQG-probability density, in particular the initial one $\rho_{o}$, to be adopted in the GLP-parameterization, see Equation (17) as well as Equations (54) and (64) above. In fact, both prescriptions should be actually regarded as mandatory prerequisites for the consistency of the GLP-representation and its ontological equivalence with the corresponding Eulerian representation of CQG-theory. In this section, we intend to show that the two issues are actually intrinsically related.

In particular we aim to prove that the initial quantum PDF can be prescribed in such a way that it coincides with a shifted Gaussian PDF, such a choice being consistent with the principle of entropy maximization (PEM), i.e., determined so to maximize the initial Boltzmann–Shannon entropy associated with the initial PDF. Consequently, the same initial PDF is shown to satisfy suitable symmetry properties (see Proposition #1). Furthermore the problem is posed of the determination of the quantum expectation values evaluated with respect to the GLP-parameterized quantum PDF. As a result, for arbitrary observables which are identified with ordinary tensor functions, equivalent representations of the GLP-quantum expectation values are pointed out (Proposition #2). A notable related implication refers to the physical interpretation of CQG-theory arising in such a context which is analogous to the so-called emergent gravity picture of quantum gravity. This follows by noting that, by suitable prescription of the initial quantum PDF, the background metric tensor $\hat{g}\left(r\left(s\right)\right)$ is uniquely determined, at any arbitrary proper-time s, in terms of an appropriate expectation value of the quantum PDF (see Proposition #3).

### 6.1. Prescription of the Stochastic PDF

The two topics indicated above actually have a unique solution. This follows at once provided the axiomatic setting of CQG-theory is invoked. Let us consider, in fact, the problem of the determination of $f(\Delta g,\hat{g})$. In the context of CQG-theory, as in the case of Quantum Mechanics (see related discussion in [22]), the independent prescription of $f(\Delta g,\hat{g})$ potentially may amount to the introduction of an additional axiom, thus possibly giving rise to additional conceptual difficulties related to the notions of quantum measurement and quantum expectation values. To overcome this issue, while leaving unaffected the axioms of CQG-theory earlier introduced in [10] and, at the same time, warranting the ontological equivalence indicated above, the only possible choice for $f(\Delta g,\hat{g})$ is that it coincides with the initial quantum PDF $\rho_{o}$. This means also, of course, that $\rho_{o}$ must be necessarily of the type (64), namely such that

(65) $$f\left(\Delta g,\hat{g}\right)\equiv \rho_{o}\left(\Delta g\pm \hat{g}\left(r_{o}\right)\right),$$

and therefore fulfilling also the constraint conditions indicated above (see Equation (46)). Incidentally, as explained below, from the conceptual viewpoint, this choice exhibits remarkable features.

### 6.2. The Initial Quantum PDF $\rho_{o}$ and Its Invariance Property

The first one, as a specific application of the GLP formalism, concerns the prescription itself of the initial quantum PDF. In fact, in validity of the identification in Equation (65), the constraints in Equation (46) included in *Requirement #1* indicated above actually uniquely prescribe the form of the initial PDF $\rho_{o}\left(\Delta g\pm \hat{g}\left(r_{o}\right)\right)$. In fact, let us introduce for definiteness the Boltzmann-Shannon entropy associated with the same PDF, which is provided by the functional

(66) $$S\left(\rho_{o}\left(\Delta g+\hat{g}\left(r_{o}\right)\right)\right)=-\int_{U_{g}}d\left(\Delta g\right)\rho_{o}\left(\Delta g+\hat{g}\left(r_{o}\right)\right)\ln\rho_{o}\left(\Delta g+\hat{g}\left(r_{o}\right)\right),$$

with $\rho_{o}\left(\Delta g,{\hat{g}}_{\mu \nu }\left(r\right)\right)\equiv f\left(\Delta g+{\hat{g}}_{\mu \nu }\left(r\right)\right)$ being assumed to satisfy the same constraint equations indicated above (i.e., Equation (46). Then, one can show that the PDF $\rho_{o}\left(\Delta g+\hat{g}\left(r_{o}\right)\right)$ which fulfills the so-called Principle of Entropy Maximization (PEM, Jaynes 1957), namely maximizes $S(\rho_{o}\left(\Delta g+\hat{g}\left(r_{o}\right)\right))$ when subject to the same constraints, is unique. Straightforward algebra shows that in the whole configuration domain $U_{g}$ it coincides with the PDF

(67) $$\rho_{o}\left(\Delta g\pm \hat{g}\left(r_{o}\right)\right)=\frac{1}{\pi^{5}r_{th}^{10}}\exp\left\{-\frac{{\left(\Delta g\pm \hat{g}\left(r_{o}\right)\right)}^{2}}{r_{th}^{2}}\right\}\equiv \rho_{G}\left(\Delta g\pm \hat{g}\left(r_{o}\right)\right),$$

with $\rho_{G}\left(\Delta g\pm \hat{g}\left(r_{o}\right)\right)$ denoting a shifted Gaussian PDF in which both $r_{th}^{2}$ and ${\left(\Delta g\pm \hat{g}\left(r_{o}\right)\right)}^{2}$ are 4-scalars, and in particular $r_{th}^{2}$ is a constant independent of (r,s), while

(68) $${\left(\Delta g\pm \hat{g}\left(r_{o}\right)\right)}^{2}\equiv {\left(\Delta g\pm \hat{g}\left(r_{o}\right)\right)}_{\mu \nu }{\left(\Delta g\pm \hat{g}\left(r_{o}\right)\right)}^{\mu \nu }.$$

Therefore, we conclude that the Gaussian PDF in Equation (67) realizes *the most likely PDF*, i.e., the one which, when subject to the constraints (46), maximizes the Boltzmann-Shannon entropy $S(\rho_{o}\left(\Delta g+\hat{g}\left(r_{o}\right)\right))$ in Equation (66).

Let us now denote with

(69) $$\rho_{G}\left(\Delta g\pm \hat{g}\left(r\right)\right)=\frac{1}{\pi^{5}r_{th}^{10}}\exp\left\{-\frac{{\left(\Delta g\pm \hat{g}\left(r\right)\right)}^{2}}{r_{th}^{2}}\right\}$$

the Gaussian PDF in Equation (67) evaluated for a generic 4-position r(s) generally different from the initial one $r_{o}\equiv r\left(s_{o}\right)$. Then, it is possible to show that a formal solution $\rho (G_{L}\left(s\right),\Delta g,s)$ of the quantum continuity equation can more generally be taken of the form

(70) $$\rho \left(G_{L}\left(s\right),\Delta g,s\right)=\rho_{G}\left(\Delta g\pm \hat{g}\left(r\right)\right)\exp\left\{-\int_{s_{o}}^{s}ds^{\prime }\frac{\partial V_{\mu \nu }\left(G_{L}\left(s^{\prime }\right),\Delta g,s^{\prime }\right)}{\partial g_{L\mu \nu }\left(s^{\prime }\right)}\right\}.$$

Let us display for this purpose an invariance property of the initial PDF. The following proposition is proven to hold.

**Proposition** **1.** **Invariance of the Gaussian PDF** $\rho_{G}\left(\Delta g\pm \hat{g}\left(r\right)\right)$

The following two propositions hold:

*P1${}_{1})$ The Gaussian PDF* $\rho_{G}\left(\Delta g\pm \hat{g}\left(r\right)\right)$ *prescribed by Equation (69) satisfies the invariance condition*

(71) $$\frac{D}{Ds}\ln\rho_{G}\left(\Delta g\pm \hat{g}\left(r\right)\right)=0.$$

*P1${}_{2})$ Equation (70) realizes a particular solution of the quantum continuity equation in Equation (57)*.

**Proof.**  To prove the invariance property in Equation (71) in proposition P1${}_{1}$, one first notices that ${\left(\Delta g\pm \hat{g}\left(r\right)\right)}^{2}\equiv {\left(\Delta g\right)}^{2}\pm 2\Delta g_{\mu \nu }{\hat{g}}^{\mu \nu }\left(r\right)+4$, where

(72) $$\left\{\begin{array}{c} {\left(\Delta g(s)\right)}^{2}={\left(\Delta g(s_{o})\right)}^{2},\\ \Delta g_{\mu \nu }\left(s\right){\hat{g}}^{\mu \nu }\left(r\right)=\Delta g_{\mu \nu }\left(s_{o}\right){\hat{g}}^{\mu \nu }\left(r\right). \end{array}\right.$$

Consequently, it follows that identically $\frac{D}{Ds}{\left(\Delta g(s)\right)}^{2}\equiv 0$, while due to the second equation in (72)

(73) $$\frac{D}{Ds}\Delta g_{\mu \nu }\left(s\right){\hat{g}}^{\mu \nu }\left(r\right)=\Delta g_{\mu \nu }\left(s_{o}\right)\frac{D}{Ds}{\hat{g}}^{\mu \nu }\left(r\right),$$

where one has that identically $\frac{D}{Ds}{\hat{g}}^{\mu \nu }\left(r\right)\equiv 0$. Hence, Equation (71) necessarily holds. This implies in turn that Equation (70) is indeed a particular solution of the quantum continuity equation, as can be easily verified by algebraic calculation after substitution in the same equation. This proves Proposition P1${}_{2}$. ☐

### 6.3. GLP-Quantum and Stochastic Expectation Values

The second implication of Equation (65) concerns the prescription of the quantum and stochastic expectation values of arbitrary observables which are identified with ordinary tensor functions.

Indeed, first, since $\Delta g\equiv \left\{\Delta g_{\mu \nu }\right\}$ is an observable, $\rho_{o}\left(\Delta g\right)$ remains in turn an observable too. Second, the *quantum expectation values* of quantum observables can be determined explicitly, *without performing a separate stochastic average*. In fact, let us consider for definiteness a generic observable which is represented by an ordinary *s*-dependent real function $X\left(s\right)\equiv X(G_{L}\left(s\right),\Delta g,s)$. According to the GLP-representation its quantum expectation value is given by the configuration-space weighted integral (hereon referred to as *GLP-quantum expectation value*):

(74) $$\langle X(s)\rangle =\int_{U_{g}}d\left(\delta G_{L}\right)\rho \left(G_{L},\Delta g,s\right)X\left(G_{L},\Delta g,s\right),$$

where the integration is performed with respect to $\delta G_{L}\equiv \delta G_{L}\left(s\right),$ keeping constant both $\delta g_{L\mu \nu }\left(s\right)$ and the background metric tensor $\hat{g}\left(r\right)\equiv$ $\hat{g}\left(r\left(s\right)\right)$ in terms of $\rho \left(G_{L},\Delta g,s\right)\equiv \rho \left(G_{L}\left(s\right),\Delta g,s\right)$, the latter being prescribed according to Equation (70). One can show that the following equivalent representations of $\langle X(s)\rangle$ hold.

**Proposition** **2.** **Equivalent representations of the GLP-quantum expectation value** $\langle X(s)\rangle$

In validity of Proposition 1 and Equation (74), the following equivalent representations of the GLP-quantum expectation value $\langle X(s)\rangle$ hold:

*(1) First, $\langle X(s)\rangle$ can be expressed by means of the expectation value in terms of the initial quantum PDF. This yields*

(75) $$\langle X(s)\rangle =\int_{U_{g}}d\left(\delta G_{L}\left(s_{o}\right)\right)\rho_{G}\left(\Delta g\pm \hat{g}\left(r\right)\right)X\left(G_{L}\left(s\right),\Delta g,s\right),$$

*where the integration is performed on the initial values of the tensor field $\delta G_{L}\left(s_{o}\right)$ instead of $\delta G_{L}\left(s\right)$. In the same integral both $\delta g_{L\mu \nu }\left(s\right)$ and $\hat{g}\left(r\left(s\right)\right)$ are again kept constant*.

*(2) Second, the same integral can also be equivalently performed in terms of the integration variable $\Delta g\equiv \left\{\Delta g_{\mu \nu }\right\}$ instead of the initial fields $\delta G_{L}\left(s_{o}\right)$, thus yielding*

(76) $$\langle X(s)\rangle =\int_{U_{g}}d\left(\Delta g\right)\rho_{o}\left(\Delta g\pm \hat{g}\left(r_{o}\right)\right)X\left(G_{L}\left(s\right),\Delta g,s\right)\equiv {\langle X\left(s\right),\hat{g}\left(r_{o}\right)\rangle }_{\Delta g},$$

*where ${\langle X\left(s\right),\hat{g}\left(r_{o}\right)\rangle }_{\Delta g}$ identifies the stochastic average of $X(G_{L}\left(s\right),\Delta g,s)$, performed in terms of the stochastic PDF $\rho_{o}\left(\Delta g\pm \hat{g}\left(r_{o}\right)\right)$ while again keeping constant $\delta g_{L\mu \nu }\left(s\right)$ and $\hat{g}\left(r\left(s\right)\right)$*.

*3) Finally, the integral in Equation (76) can also be equivalently performed in terms of the integral*

(77) $$\langle X(s)\rangle =\int_{U_{g}}d\left(\Delta g\right)\rho_{o}\left(\Delta g\pm \hat{g}\left(r\left(s\right)\right)\right)X\left(G_{L}\left(s\right),\Delta g,s\right)\equiv {\langle X\left(s\right),\hat{g}\left(r\left(s\right)\right)\rangle }_{\Delta g},$$

*where ${\langle X\left(s\right),\hat{g}\left(r\left(s\right)\right)\rangle }_{\Delta g}$ identifies the stochastic average of $X(G_{L}\left(s\right),\Delta g,s),$ performed in terms of the stochastic PDF $\rho_{o}\left(\Delta g\pm \hat{g}\left(r\right)\right)$ while keeping constant $\delta g_{L\mu \nu }\left(s\right)$ and $\hat{g}\left(r\right)$*.

**Proof.**  Consider first Equation (75). Its proof follows by noting that the integral in Equation (74) can be equivalently represented in terms of the inverse mapping $\delta G_{L}\left(s\right)\to \delta G_{L}\left(s_{o}\right)$. This implies, in fact, the differential identity

(78) $$d\left(\delta G_{L}\left(s\right)\right)=d\left(\delta G_{L}\left(s_{o}\right)\right)\left|\frac{\partial \delta G_{L}\left(s\right)}{\partial \delta G_{L}\left(s_{o}\right)}\right|,$$

where, thanks to Liouville theorem the Jacobian determinant $\left|\frac{\partial \delta G_{L}\left(s\right)}{\partial \delta G_{L}\left(s_{o}\right)}\right|$ can be shown to be

(79) $$\left|\frac{\partial \delta G_{L}\left(s\right)}{\partial \delta G_{L}\left(s_{o}\right)}\right|=\exp\left\{\int_{s_{o}}^{s}ds^{\prime }\frac{\partial V_{\mu \nu }\left(G_{L}\left(s^{\prime }\right),\Delta g,s^{\prime }\right)}{\partial g_{L\mu \nu }\left(s^{\prime }\right)}\right\}.$$

Next, by invoking the solution of the quantum continuity Equation (70), conservation of probability warrants that

(80) $$d\left(\delta G_{L}\right)\rho \left(G_{L}\left(s\right),\Delta g,s\right)=d\left(\delta G_{L}\left(s_{o}\right)\right)\rho_{o}\left(\Delta g\pm \hat{g}\left(r\left(s\right)\right)\right),$$

which in turn implies Equation (75). The proof of Equation (76) is obtained in a similar way by noting that (see Equation (43)) $\Delta g_{\mu \nu }=\delta g_{L\mu \nu }\left(s_{o}\right)-\delta G_{L\mu \nu }\left(s_{o}\right)$ so that the same integral (75) can also be equivalently performed in terms of the integration variable $\Delta g\equiv \left\{\Delta g_{\mu \nu }\right\}$ while keeping constant $\delta g_{L\mu \nu }\left(s_{o}\right)$ and $\hat{g}\left(r\right)$. Hence, it follows that

(81) $$d\left(\delta G_{L}\left(s_{o}\right)\right)=d\left(\Delta g\right)\left|\frac{\partial \delta G_{L}\left(s_{o}\right)}{\partial \Delta g}\right|=d\left(\Delta g\right),$$

since the Jacobian determinant $\left|\frac{\partial \delta G_{L}\left(s_{o}\right)}{\partial \Delta g}\right|$ is by construction identically equal to 1. Hence, the differential identity (80) necessarily holds, thus yielding also Equation (76). Finally, the proof of Equation (77) follows from Equation (76) being an immediate consequence of Proposition 1. ☐

### 6.4. Generalized Gaussian PDF and Emergent Gravity Interpretation

Let us examine the implications of the previous Propositions 1 and 2. The first one concerns the determination of the proper-time evolved quantum PDF $\rho (G_{L}\left(s\right),\Delta g,s)$, to be based on Proposition 1 (see the conservation Equation (71)) and Equation (63). This is given by Equation (70). Notice that, although $\rho_{G}\left(\Delta g\pm \hat{g}\left(r\right)\right)$ is a shifted Gaussian PDF, $\rho (G_{L}\left(s\right),\Delta g,s)$ is generally not so. Its precise realization depends in fact on the quantum phase-function $S^{\left(q\right)}\left(G_{L}\left(s\right),\Delta g,s\right)$, i.e., the corresponding solution of the quantum H-J equation (in Equation (57)). As a result, the tensor velocity field $V_{\mu \nu }\left(G_{L}\left(s\right),\Delta g,s\right)$ at this stage is still unknown, thus leaving still undetermined the precise functional form of $\rho (G_{L}\left(s\right),\Delta g,s),$ so that in general the proper-time evolved PDF $\rho (G_{L}\left(s\right),\Delta g,s),$ in contrast to the initial PDF, may be generally not Gaussian any more. For this reason, Equation (70) will be referred to in the following as *Generalized Gaussian PDF*.

The second implication, which is also relevant for the physical interpretation of the GLP-approach, concerns the following statement.

**Proposition** **3.** **Determination of** $\hat{g}\left(r\right)$ **(Emergent gravity)**

*The generalized Gaussian PDF (70) for all $r\in \left\{Q^{4},\hat{g}\right\}$ admits for the stochastic displacement* 4-*tensor $\Delta g_{\mu \nu }$ the following GLP-quantum/stochastic expectation value (in which both $\delta g_{L\mu \nu }\left(s\right)$ and $\hat{g}\left(r\left(s\right)\right)$ are again kept constant in the integration):*

(82) $$\langle \Delta g_{\mu \nu }\rangle \equiv {\langle \Delta g_{\mu \nu }\rangle }_{\Delta g}=\int_{U_{g}}d\left(\Delta g\right)\rho_{G}\left(\Delta g\pm \hat{g}\left(r\right)\right)\Delta g_{\mu \nu }=\mp {\hat{g}}_{\mu \nu }\left(r\right).$$

**Proof.**  The proof follows as an immediate consequence of Proposition 2 and in particular thanks to Equation (77). ☐

The consequence is that, in the whole space-time and for all proper-times *s* (i.e., for arbitrary $\left(r\equiv r(s),s\right)$), the local value of the background metric tensor $\hat{g}\left(r\right)$ is prescribed by means of the GLP-quantum expectation value of the stochastic displacement 4-tensor $\Delta g_{\mu \nu }$, i.e., $\langle \Delta g_{\mu \nu }\rangle$, or equivalently by means of the corresponding stochastic average ${\langle \Delta g_{\mu \nu }\rangle }_{\Delta g}$ evaluated in terms of the stochastic PDF $\rho_{G}\left(\Delta g\pm \hat{g}\left(r\right)\right)$. In this regard one notices that for the validity of Proposition 3 the initial PDF must be identified with the stochastic PDF $f\left(\Delta g,\hat{g}\right)$, with the latter satisfying the constraint conditions (46). This implies the existence of an emergent gravity phenomenon, in the sense that the background metric tensor $\hat{g}\left(r\right)\equiv \hat{g}\left(r\left(s\right)\right)$ “emerges” from the quantum gravitational field $g_{\mu \nu }$ as the quantum/stochastic expectation value of the stochastic quantum displacement tensor $\Delta g_{\mu \nu }$ which characterizes the covariant GLP theory.

The conclusion provides a physical interpretation of CQG-theory. Indeed, consistent with the second-type emergent-gravity paradigm referred to above (see Introduction), the background space-time appears through a mean-field gravitational tensor as the result of a suitable ensemble average of an underlying quantum/stochastic virtual space-time whose quantum-wave dynamics is described by GLP trajectories. A notable aspect of the conclusion is, however, that the representation of the proper-time evolved PDF provided by Equation (70) is of general character. In fact, Equation (82) holds independent also of the precise prescription of the classical/quantum effective potential in the quantum Hamiltonian operator. Therefore, *the emergent-gravity interpretation of* $\hat{g}\left(r\right)$ *is an intrinsic characteristic feature of the GLP-representation* developed here for CQG-theory, whereby the background metric tensor $\hat{g}\left(r\right)$ can be effectively interpreted as arising from the stochastic fluctuations of GLP trajectories having a suitable stochastic probability distribution identified with a Gaussian or more generally Gaussian-like PDF. It follows that $\hat{g}\left(r\right)$ can be then obtained exactly as a statistical moment in terms of weighted integral over the stochastic tensor $\Delta g_{\mu \nu }$.

In this sense, the concept of emergent gravity proposed here has similarities with the analogous one to be found in the literature, namely the conjecture that the geometrical properties of space-time should reveal themselves as a mean field description of microscopic stochastic or quantum degrees of freedom underlying the classical solution [75,76]. However, the physical context proposed here differs from the customary one adopted in the literature, whereby according to the common emergent gravity paradigm the Einstein field equations of gravity should have an emergent character in that, in validity of suitable assumptions, they can be shown to arise from a thermodynamic approach to space-time [77,78]. Nevertheless, the explicit construction of particular solutions of the GLP-parameterized quantum continuity and H-J equations indicated above (see Equation (57)) remains necessary and requires the introduction of suitable representations both for the quantum phase-function $S^{\left(q\right)}\left(G_{L}\left(s\right),\Delta g,s\right)$ and the quantum effective potential $V(G_{L}\left(s\right),\Delta g,s)$ (see next Section).

## 7. GLP Approach: Polynomial Decomposition of the Quantum Phase Function

Based on these premises, we can now implement the GLP formalism and proceed constructing particular solutions of the quantum H-J equation (see second part of Equation (57)). More precisely, the goal here is to look for solutions of the quantum phase function expressed in the GLP-parameterization, i.e., $S^{\left(q\right)}\left(G_{L}\left(s\right),\Delta g,s\right),$ which are expressed by means of polynomial decompositions in terms of power series of the stochastic tensor Δg. For definiteness, in the sequel the case is considered in which the following pre-requisites apply:

(A) “Harmonic” polynomial decomposition of $S^{\left(q\right)}\left(G_{L}\left(s\right),\Delta g,s\right)$, i.e., the same quantum-phase function is expressed in terms of a second-degree polynomial of the form

(83) $$S^{\left(q\right)}\left(G_{L}\left(s\right),\Delta g,s\right)=\frac{a_{pq}^{\alpha \beta }\left(s\right)}{2}\Delta g_{\alpha \beta }\Delta g^{pq}+b_{\alpha \beta }\left(s\right)\Delta g^{\alpha \beta }+c\left(s\right),$$

with $a_{\mu \nu }^{\alpha \beta }\left(s\right)$, $b_{\mu \nu }\left(s\right)$ and c(s) denoting, respectively, suitable real 4-tensors and a 4-scalar functions of *s* to be determined in terms of the same H-J equation. As shown below, this implies that the effective kinetic energy $T(G_{L}\left(s\right),\Delta g,s)$ defined by Equation (24) and the Bohm effective quantum potential $V_{QM}\left(G_{L}\left(s\right),\Delta g,s\right)$ prescribed according to Equation (25) are both realized by means of polynomials of second degree in Δg.

(B) An analogous “Harmonic” polynomial decomposition holds for $V(G_{L}\left(s\right),\Delta g,s)$: namely, that a polynomial representation of analogous type should apply also for the total quantum effective potential density appearing in the quantum H-J equation (see Equation (19)). The latter, to be generally considered of the form $V(G_{L}\left(s\right),\Delta g,s)$, should therefore admit a polynomial representation of the type

(84) $$V\left(G_{L}\left(s\right),\Delta g,s\right)=\frac{A_{pq}^{\alpha \beta }\left(s\right)}{2}\Delta g_{\alpha \beta }\Delta g^{pq}+B_{\alpha \beta }\left(s\right)\Delta g^{\alpha \beta }+C\left(s\right),$$

where the tensor coefficients $A_{\mu \nu }^{\alpha \beta }\left(s\right)$, $B_{\mu \nu }\left(s\right)$ and C(s) are considered here functions of *s* alone to be suitably determined.

### 7.1. Implications of the Polynomial Decomposition for $S^{\left(q\right)}\left(G_{L}\left(s\right),\Delta g,s\right)$

Let us investigate in detail the consequences of the prescription (83) set on the quantum phase-function $S^{\left(q\right)}\left(G_{L}\left(s\right),\Delta g,s\right)$. One notices, first, that this property permits to identify uniquely the proper-time evolved quantum PDF in terms of a Gaussian PDF, which means that, apart for a proper-time dependent factor, in such a case the PDF $\rho (G_{L}\left(s\right),\Delta g,s)$ becomes intrinsically non-dispersive in character. In this regard the following statement holds.

**Proposition** **4.** **Determination of the Gaussian PDF** $\rho (G_{L}\left(s\right),\Delta g,s)$

*In validity of the harmonic polynomial decomposition in Equation (83), the generalized Gaussian PDF in Equation (70) takes the form of the Gaussian PDF*

(85) $$\rho \left(G_{L}\left(s\right),\Delta g,s\right)\equiv \rho_{G}\left(\Delta g+\hat{g}\left(r\right)\right)\exp\left\{-16\int_{s_{o}}^{s}dsp^{2}\left(s^{\prime }\right)\frac{a(s^{\prime })}{\alpha L}\right\},$$

*where $p(s^{\prime })$ and $a(s^{\prime })$ are the* 4-*scalar functions respectively prescribed by Equation (A19) and Equation (A18) in Appendix A*.

**Proof.**  The proof follows by noting that in this case the tensor velocity $V_{\mu \nu }\left(G_{L}\left(s\right),\Delta g,s\right)$ defined by Equation (48) becomes explicitly

(86) $$V^{\mu \nu }\left(G_{L}\left(s\right),\Delta g,s\right)\equiv \frac{1}{\alpha L}\frac{\partial S^{\left(q\right)}\left(G_{L}\left(s\right),\Delta g,s\right)}{\partial g_{L\mu \nu }\left(s\right)}=\frac{a_{pq}^{\alpha \beta }\left(s\right)}{\alpha L}\frac{\partial \Delta g_{\alpha \beta }}{\partial g_{L\mu \nu }\left(s\right)}\Delta g^{pq}+\frac{1}{\alpha L}\frac{\partial \Delta g_{\alpha \beta }}{\partial g_{L\mu \nu }\left(s\right)}b^{\alpha \beta }\left(s\right).$$

Consequently, the divergence of the tensor velocity $\frac{\partial V^{\mu \nu }\left(\Delta g,s^{\prime }\right)}{\partial g_{L}^{\mu \nu }\left(s^{\prime }\right)}$, which enters the exponential occurring on the R.H.S. of Equation (63), delivers

(87) $$\frac{\partial V^{\mu \nu }\left(\Delta g,s^{\prime }\right)}{\partial g_{L}^{\mu \nu }\left(s^{\prime }\right)}=\frac{1}{\alpha L}\frac{\partial^{2}S^{\left(q\right)}\left(\Delta g,s^{\prime }\right)}{\partial g_{L}^{\mu \nu }\left(s^{\prime }\right)\partial g_{L\mu \nu }\left(s^{\prime }\right)}=\frac{a_{pq}^{\alpha \beta }\left(s\right)}{\alpha L}\frac{\partial \Delta g_{\alpha \beta }}{\partial g_{L\mu \nu }\left(s^{\prime }\right)}\frac{\partial \Delta g^{pq}}{\partial g_{L}^{\mu \nu }\left(s^{\prime }\right)},$$

where the evaluation of the fourth order tensor $\frac{\partial \Delta g_{\alpha \beta }}{\partial g_{L\mu \nu }\left(s\right)}$ is reported in Appendix A (see, e.g., Equation (A2) together with Propositions (A1) and (A2)).

Hence, the previous equation implies in turn

(88) $$\frac{\partial V^{\mu \nu }\left(\Delta g,s\right)}{\partial g_{L}^{\mu \nu }\left(s\right)}=p^{2}\left(s\right)\frac{a_{pq}^{\alpha \beta }\left(s\right)}{\alpha L}\delta_{\alpha \beta }^{\mu \nu }\delta_{\mu \nu }^{pq}\equiv 16p^{2}\left(s\right)\frac{a(s)}{\alpha L},$$

where the notation $\delta_{\alpha \beta }^{\mu \nu }\equiv \delta_{\alpha }^{\mu }\delta_{\beta }^{\nu }$ has been introduced. Consequently, the proper-time evolved quantum PDF in Equation (70) takes the form of Equation (85). ☐

Next, let us consider the evaluation of effective kinetic energy $T(G_{L}\left(s\right),\Delta g,s)$ defined by Equation (24) and of the Bohm potential given by Equation (25). Regarding $T(G_{L}\left(s\right),\Delta g,s),$ thanks again to Equation (83), direct evaluation delivers

(89) $$T\left(G_{L}\left(s\right),\Delta g,s\right)=\frac{p^{2}\left(s\right)}{2\alpha L}\left[a_{\mu \nu }^{\alpha \beta }\left(s\right)a_{pq}^{\mu \nu }\left(s\right)\Delta g_{\alpha \beta }\Delta g^{pq}+b_{\mu \nu }\left(s\right)b^{\mu \nu }\left(s\right)+2a_{\alpha \beta }^{\mu \nu }\left(s\right)b_{\mu \nu }\left(s\right)\Delta g^{\alpha \beta }\right].$$

Concerning instead the Bohm potential, one notices that, by invoking Proposition 4 (i.e., Equation (85)), the two source terms on the R.H.S. of Equation (25) become, respectively,

(90) $$\begin{array}{ccc} \frac{\partial \ln\rho (G_{L}\left(s\right),\Delta g,s)}{\partial g_{L}^{\mu \nu }\left(s\right)} & = & -\frac{2}{r_{th}^{2}}p\left(s\right)\left(\Delta g_{\mu \nu }\pm {\hat{g}}_{\mu \nu }\left(r\right)\right), \end{array}$$

(91) $$\begin{array}{ccc} \frac{\partial^{2}\ln\rho \left(G_{L}\left(s\right),\Delta g,s\right)}{\partial g_{L\mu \nu }\left(s\right)\partial g_{L}^{\mu \nu }\left(s\right)} & = & -\frac{8}{r_{th}^{2}}p^{2}\left(s\right). \end{array}$$

Consequently, direct substitution in the same equation delivers for the Bohm potential the representation:

(92) $$\begin{array}{ccc} V_{QM}\left(G_{L}\left(s\right),\Delta g,s\right) & \equiv & -\frac{\hbar^{2}}{8\alpha L}\left[\frac{2}{r_{th}^{2}}p\left(s\right)\left(\Delta g_{\mu \nu }\pm {\hat{g}}_{\mu \nu }\left(r\right)\right)\right]\left[\frac{2}{r_{th}^{2}}p\left(s\right)\left(\Delta g^{\mu \nu }\pm {\hat{g}}^{\mu \nu }\left(r\right)\right)\right]\\ & & -\frac{\hbar^{2}}{4\alpha L}\left[-\frac{8}{r_{th}^{2}}p^{2}\left(s\right)\right], \end{array}$$

which can be equivalently written as

(93) $$V_{QM}\left(G_{L}\left(s\right),\Delta g,s\right)\equiv -\frac{\hbar^{2}p^{2}\left(s\right)}{2\alpha Lr_{th}^{4}}\left(\Delta g_{\mu \nu }\Delta g^{\mu \nu }\pm 2{\hat{g}}_{\mu \nu }\left(r\right)\Delta g^{\mu \nu }+4\right)+\frac{2\hbar^{2}p^{2}\left(s\right)}{\alpha Lr_{th}^{2}}.$$

### 7.2. Implications of the Polynomial Decomposition for $V(G_{L}\left(s\right),\Delta g,s)$

That an explicit realization of the polynomial representation of the type in Equation (84) is actually possible for the effective classical potential density $V(G_{L}\left(s\right),\Delta g,s)$ given by Equation (19) follows by its definition. For definiteness, let us show how this task can be achieved for a specific realization, i.e., in the case of vacuum. The following proposition holds.

**Proposition** **5.** **Harmonic representation of the vacuum effective potential**

*The vacuum effective potential in Equation (19) in the harmonic polynomial representation in Equation (84) takes the form*

(94) $$V_{o}\left(g+\Delta g\right)=2\sigma \alpha L\Lambda +\sigma \alpha L\Lambda \left[-\frac{1}{2}\Delta g_{\mu \nu }\Delta g^{\mu \nu }-\frac{1}{2}\Delta g^{\mu \nu }{\hat{g}}_{\mu \nu }\left(r\right)\Delta g^{\alpha \beta }{\hat{g}}_{\alpha \beta }\left(r\right)\right].$$

**Proof.**  In fact, from Equation (61), the vacuum effective potential $V_{o}\left(\hat{g}+\Delta g\right)$ becomes

(95) $$V_{o}\left(g+\Delta g\right)\equiv \sigma \alpha L\Lambda \left[2-\frac{1}{4}\left({\hat{g}}_{\mu \nu }\left(r\right)+\Delta g_{\mu \nu }\right)\left({\hat{g}}^{\mu \nu }\left(r\right)+\Delta g^{\mu \nu }\right)\right]\left[\left({\hat{g}}_{pq}\left(r\right)+\Delta g_{pq}\right){\hat{g}}^{pq}\left(r\right)-2\right].$$

The harmonic representation is obtained dropping terms of order ${\left(\Delta g\right)}^{3}$ or higher. When this is done in the previous equation, Equation (94) is recovered at once. ☐

The form of the source term in Equation (94) suggests to seek for the tensor coefficient $a_{\mu \nu }^{\alpha \beta }\left(s\right)$ in Equation (83) a particular realization of the form

(96) $$a_{pq}^{\alpha \beta }\left(s\right)=\frac{1}{2}\left[a_{\left(o\right)}\left(s\right)\delta_{pq}^{\alpha \beta }+a_{\left(1\right)}\left(s\right){\hat{g}}_{pq}\left(r\right){\hat{g}}^{\alpha \beta }\left(r\right)\right],$$

so that upon invoking Equation (A18), namely letting $a_{\mu \nu }^{\alpha \beta }\left(s\right)\delta_{\alpha \beta }^{\mu \nu }\equiv 4a\left(s\right)$, it follows $a\left(s\right)=\frac{1}{2}\left[a_{\left(o\right)}+a_{\left(1\right)}\right]$. Consequently, one finds that the tensor coefficients $a_{pq}^{\alpha \beta }\left(s\right)$ in Equation (83) can also be written as

(97) $$a_{pq}^{\alpha \beta }\left(s\right)=\frac{1}{2}\left[2a\left(s\right)\delta_{pq}^{\alpha \beta }+a_{\left(1\right)}\left(s\right)\left({\hat{g}}_{pq}\left(r\right){\hat{g}}^{\alpha \beta }\left(r\right)-\delta_{pq}^{\alpha \beta }\right)\right].$$

In addition, straightforward algebra yields the identities represented by Equations (A23)–(A29) which are reported in Appendix B.

### 7.3. Construction of the GLP-Equations

We now pose the problem of the construction of the set of ODEs, which, in validity of the Harmonic polynomial decompositions indicated above, determine a separable solution of the quantum H-J equation in Equation (57), and are thus equivalent to the same equation. In the case of the vacuum effective potential by equating all terms in the polynomial expansion, one obtains a set of ODEs for the 4-scalar coefficients $a_{\left(o\right)}\left(s\right)$, $a_{\left(1\right)}\left(s\right)$ and c(s) and the 4-tensor $b_{\alpha \beta }\left(s\right)$, here referred to as *GLP-equations*. These are provided by the first-order ODEs:

(98) $$\left\{\begin{array}{c} \frac{1}{4}\frac{d}{ds}a_{\left(o\right)}\left(s\right)=\frac{p^{2}\left(s\right)}{8\alpha L}a_{\left(o\right)}^{2}\left(s\right)-\frac{\hbar^{2}}{2\alpha L}\frac{1}{r_{th}^{4}}p^{2}\left(s\right)+\frac{1}{2}\sigma \alpha L\Lambda +G_{\left(o\right)},\\ \frac{1}{4}\frac{d}{ds}a_{\left(1\right)}\left(s\right)=\frac{p^{2}\left(s\right)}{8\alpha L}\left(4a_{\left(1\right)}^{2}\left(s\right)+2a_{\left(o\right)}\left(s\right)a_{\left(1\right)}\left(s\right)\right)+\frac{1}{2}\sigma \alpha L\Lambda +G_{\left(1\right)},\\ \frac{d}{ds}b_{\alpha \beta }\left(s\right)=\frac{p^{2}\left(s\right)}{2\alpha L}\left[b_{\alpha \beta }a_{\left(o\right)}\left(s\right)+a_{\left(1\right)}\left(s\right){\hat{g}}_{\alpha \beta }\left(r\right){\hat{g}}^{\mu \nu }\left(r\right)b_{\mu \nu }\left(s\right)\right],\\ \frac{d}{ds}c\left(s\right)=\frac{p^{2}\left(s\right)}{2\alpha L}b_{\mu \nu }\left(s\right)b^{\mu \nu }\left(s\right)+\frac{2\hbar^{2}}{\alpha L}\frac{1}{r_{th}^{2}}p^{2}\left(s\right)+C_{o}\left(s\right), \end{array}\right.$$

where $G_{\left(o\right)}$, $G_{\left(1\right)}$ and $C_{o}\left(s\right)$ are in principle arbitrary 4-scalar gauge functions. These can be prescribed in such a way that there exists a stationary null solution for the 4-scalar coefficient $a\left(s\right)\equiv \hat{a}\left(s\right)$, namely such that for all s∈I, $\hat{a}\left(s\right)=0$, and hence identically for all $s,s_{o}\in I$,

(99) $$\left\{\begin{array}{c} {\hat{a}}_{\left(o\right)}\left(s\right)\equiv {\hat{a}}_{\left(o\right)}\left(s_{o}\right),\\ {\hat{a}}_{\left(1\right)}\left(s\right)={\hat{a}}_{\left(1\right)}\left(s_{o}\right),\\ {\hat{a}}_{\left(1\right)}\left(s_{o}\right)=-{\hat{a}}_{\left(o\right)}\left(s_{o}\right), \end{array}\right.$$

which realizes a particular stationary solution of Equation (98). This requires suitably-identifying the gauge functions $G_{\left(o\right)}$ and $G_{\left(1\right)}$, which, for consistency with Equation (99), can always be prescribed in such a way that

(100) $$\left\{\begin{array}{c} G_{\left(o\right)}=-\frac{1}{8\alpha L}{\hat{a}}_{\left(o\right)}^{2}\left(s_{o}\right)+\frac{\hbar^{2}}{2\alpha L}\frac{1}{r_{th}^{4}}-\frac{1}{2}\sigma \alpha L\Lambda ,\\ G_{\left(1\right)}=-\frac{1}{4\alpha L}{\hat{a}}_{\left(o\right)}^{2}\left(s_{o}\right)-\frac{1}{2}\sigma \alpha L\Lambda \equiv 0, \end{array}\right.$$

so that the first two parts of Equation (98) can be written explicitly as

(101) $$\left\{\begin{array}{c} \frac{1}{4}\frac{d}{ds}a_{\left(o\right)}\left(s\right)=\frac{1}{8\alpha L}\left[p^{2}\left(s\right)a_{\left(o\right)}^{2}\left(s\right)-2\alpha^{2}L^{2}\Lambda \right]-\frac{\hbar^{2}}{2\alpha L}\frac{1}{r_{th}^{4}}\left[p^{2}\left(s\right)-1\right],\\ \frac{1}{4}\frac{d}{ds}a_{\left(1\right)}\left(s\right)=\frac{p^{2}\left(s\right)}{8\alpha L}\left(4a_{\left(1\right)}^{2}\left(s\right)+2a_{\left(o\right)}\left(s\right)a_{\left(1\right)}\left(s\right)\right)-\frac{1}{2}\alpha L\Lambda . \end{array}\right.$$

We finally notice that the previous equations can also be conveniently cast in dimensionless form. Noting that $\left[a\right]=\left[a_{\left(o\right)}\right]=\left[a_{\left(1\right)}\right]=\left[\hbar \right]=\left[\alpha \right]$, the dimensionless representation is obtained by means of the dimensionless variables

(102) $$\left\{\begin{array}{c} {\bar{a}}_{\left(o\right)}\left(\theta \right)=\frac{a_{\left(o\right)}\left(\theta \right)}{\alpha },\\ {\bar{a}}_{\left(1\right)}\left(\theta \right)=\frac{a_{\left(1\right)}}{\alpha },\\ {\bar{b}}_{\alpha \beta }\left(\theta \right)=\frac{b_{\alpha \beta }\left(s\right)}{\alpha },\\ \bar{c}\left(\theta \right)=\frac{c}{\alpha },\\ \theta =\frac{2s}{L},\\ \bar{\Lambda }=\Lambda L^{2}≅9.408, \end{array}\right.$$

where $\bar{\Lambda }$ identifies in dimensionless units the experimental value of cosmological constant, here evaluated in terms of the Compton Length *L* which corresponds to the graviton-mass estimate given in [10]. Then, introducing the notations

(103) $$\left\{\begin{array}{c} Y\left(\theta \right)\equiv {\left(1+\int_{\theta_{o}}^{\theta }d\theta^{\prime }\bar{a}\left(\theta^{\prime }\right)\right)}^{1/2},\\ Z\left(\theta \right)=\frac{{\bar{a}}_{\left(1\right)}\left(\theta \right)}{Y{\left(\theta \right)}^{2}}, \end{array}\right.$$

Equation (98) can be shown to be equivalent to the following set of ODEs for the coefficients $\bar{a}\left(\theta \right)$ and ${\bar{a}}_{\left(1\right)}\left(\theta \right)$:

(104) $$\left\{\begin{array}{c} \frac{d^{2}}{d\theta^{2}}Y\left(\theta \right)=\frac{3}{16}\frac{Z^{2}\left(\theta \right)}{Y(\theta )}-\frac{3}{16}\frac{\bar{\Lambda }}{Y(\theta )}+\frac{\hbar^{2}}{4\alpha^{2}r_{th}^{4}}\frac{Y{\left(\theta \right)}^{2}-1}{Y{\left(\theta \right)}^{3}},\\ \frac{d}{d\theta }Z\left(\theta \right)=\frac{Z^{2}\left(\theta \right)}{Y(\theta )}-\frac{1}{2}\frac{\bar{\Lambda }}{Y(\theta )}, \end{array}\right.$$

which admit the stationary solution

(105) $$\left\{\begin{array}{c} \bar{a}\left(s\right)=0,\\ {\bar{a}}_{\left(o\right)}^{2}\left(s\right)-\frac{1}{2}\bar{\Lambda }=0. \end{array}\right.$$

### 7.4. Small-Amplitude Solutions: Conditions of Validity

Now, we look for small-amplitude solutions of Equation (104). For definiteness, let us introduce the representations

(106) $$\left\{\begin{array}{c} Y\left(\theta \right)=Y\left(\theta_{o}\right)+\delta Y\left(\theta \right),\\ Z\left(\theta \right)=Z\left(\theta_{o}\right)+\delta Z\left(\theta \right), \end{array}\right.$$

with $Y(\theta_{o})=1$, $Z\left(\theta_{o}\right)={\bar{a}}_{\left(1\right)}\left(\theta_{o}\right)=\pm \sqrt{\frac{1}{2}\bar{\Lambda }}$ and δY(θ), δZ(θ) denoting displacements such that for all $\theta \in I_{s_{\theta o}}^{\left(+\right)}\equiv \left[\theta_{o},+\infty \right]$

(107) $$\begin{array}{ccc} 0 & < & \delta Y(\theta )\ll 1,\\ 0 & < & \left|\delta Z\left(\theta \right)/\sqrt{\frac{1}{2}\bar{\Lambda }}\right|\ll 1. \end{array}$$

These will be denoted as small-amplitude solutions. In this regard, the following proposition holds.

**Proposition** **6.** **Small-amplitude solutions of Equation (104)**

*For all $s\in I_{s_{o}}^{\left(+\right)}\equiv \left[s_{o},+\infty \right]$ Equation (104) admit small-amplitude solutions*.

**Proof.**  In fact, upon linearization, Equation (104) implies

(108) $$\left\{\begin{array}{c} \frac{d^{2}}{d\theta^{2}}\delta Y\left(\theta \right)=\frac{3}{16}\left[\pm 2\sqrt{\frac{1}{2}\bar{\Lambda }}\delta Z-\frac{1}{2}\bar{\Lambda }\delta Y\left(\theta \right)\right]+\frac{3}{16}\bar{\Lambda }\delta Y\left(\theta \right)+\frac{\hbar^{2}}{4\alpha^{2}r_{th}^{4}}2\delta Y\left(\theta \right),\\ \frac{d}{d\theta }\delta Z\left(\theta \right)=\pm 2\sqrt{\frac{1}{2}\bar{\Lambda }}\delta Z. \end{array}\right.$$

The two equations deliver, respectively, the solutions

(109) $$\left\{\begin{array}{c} \delta Z\left(\theta \right)=\delta Z\left(\theta_{o}\right)\exp\left\{\pm 2\sqrt{\frac{1}{2}\bar{\Lambda }}\left(\theta -\theta_{o}\right)\right\},\\ \delta a\left(\theta \right)=A\delta Z\left(\theta_{o}\right)\exp\left\{\pm 2\sqrt{\frac{1}{2}\bar{\Lambda }}\left(\theta -\theta_{o}\right)\right\}, \end{array}\right.$$

with *A* denoting the constant coefficient

(110) $$A=\frac{\frac{3}{4}\bar{\Lambda }}{1-\frac{9}{32}\bar{\Lambda }-\frac{\hbar^{2}}{2\alpha^{2}r_{th}^{4}}}.$$

Consequently, Equation (109) implies also that

(111) $$\delta a_{\left(1\right)}\left(\theta \right)=\delta Z\left(\theta \right)\left[1+\frac{A}{2}\right].$$

 ☐

Thus, we conclude that small-amplitude solutions of the GLP-equations in Equation (104) indeed exist which depend exponentially on proper time, the exponential factor being of the form $\exp\left\{-2\sqrt{\frac{1}{2}\bar{\Lambda }}\left(\theta -\theta_{o}\right)\right\}$ or $\exp\left\{2\sqrt{\frac{1}{2}\bar{\Lambda }}\left(\theta -\theta_{o}\right)\right\}$, respectively. These are referred to, respectively, as *decay* and *blow-up* small-amplitude solutions. In the two cases for $\theta -\theta_{o}\to +\infty$, these either decay to the constant solution or diverge exponentially. Therefore, quantum stationary solutions can be identified with asymptotic ones, i.e., as final states of decaying quantum solutions. Blow-up solutions, however, for finite times $\theta -\theta_{o}>0$ necessarily violate the ordering assumptions (107) and, as such, Equations (109) and (111) are no longer applicable in such a case.

The investigation of the blow-up solutions requires therefore the proper consideration of the set of GLP-equations in Equation (104). One can show, however, that, if the following asymptotic orderings apply,

(112) $$\begin{array}{ccc} Y(\theta ) & \gg & 1, \end{array}$$

(113) $$\begin{array}{ccc} \left|Z\left(\theta \right)/\sqrt{\frac{1}{2}\bar{\Lambda }}\right| & \gg & 1, \end{array}$$

then, in such a case, the asymptotic limits must apply

(114) $$\begin{array}{ccc} \lim_{\theta -\theta_{o}\to +\infty }Y\left(\theta \right) & = & +\infty , \end{array}$$

(115) $$\begin{array}{ccc} \lim_{\theta -\theta_{o}\to +\infty }\frac{d}{d\theta }Y\left(\theta \right) & = & 0. \end{array}$$

These imply in turn also the vanishing of the 4-scalar coefficient p(s) (see Appendix A) in the proper-time limit $s-s_{o}\to +\infty$, i.e.,

(116) $$\lim_{s-s_{o}\to +\infty }p\left(s\right)=0.$$

The implication of Equation (116) is however the violation in the same limit of the Heisenberg inequality

(117) $$\langle {\left(\Delta g_{{}_{\left(\mu )(\nu \right)}}\right)}^{2}\rangle {\langle {\left(\Delta \pi_{\mu \nu }\right)}^{2}\rangle }_{1}\geq \frac{\hbar^{2}}{4},$$

pointed out in [11], with $\langle {\left(\Delta g_{{}_{\left(\mu )(\nu \right)}}\right)}^{2}\rangle$ and ${\langle {\left(\Delta \pi_{\mu \nu }\right)}^{2}\rangle }_{1}$ denoting respectively

(118) $$\begin{array}{c} \left.{\langle {\left(\Delta \pi_{{}_{\mu \nu }}\right)}^{2}\rangle }_{1}=\frac{\hbar^{2}}{4}\int_{U_{g}}d\left(g\right)\rho \frac{\partial \ln\rho }{\partial g^{\mu \nu }}\frac{\partial \ln\rho }{\partial g^{\left(\mu )(\nu \right)}},\right. \end{array}$$

(119) $$\begin{array}{ccc} \langle {\left(\Delta g_{\mu \nu }\right)}^{2}\rangle & = & \int_{U_{g}}d\left(g\right)\rho \left(g_{\mu \nu }-{\tilde{g}}_{\mu \nu }\right)\left(g_{\left(\mu )(\nu \right)}-{\tilde{g}}_{\left(\mu )(\nu \right)}\right)=\frac{1}{10}r_{th}^{2}. \end{array}$$

In fact, due to Equation (116), it follows that

(120) $$\lim_{s-s_{o}\to +\infty }\langle {\left(\Delta g_{\mu \nu }\right)}^{2}\rangle =0.$$

Instead, one can show that constant or small-amplitude decaying solutions satisfy the Heisenberg inequality in Equation (117) and as such realize physically admissible quantum solutions. Such a conclusion, therefore, rules out blow-up solutions from the class of physically-admissible solutions in the same limit.

## 8. Conclusions

In this paper, the basic principles of a new trajectory-based approach to manifestly-covariant quantum gravity (CQG) theory have been laid down. This provide new physical insight into the nature and behavior of the manifestly-covariant quantum-wave equation and corresponding equivalent set of quantum hydrodynamic equations that are realized by means of CQG-theory. For its similarity with the analogous Generalized Lagrangian Path approach holding in non relativistic quantum mechanics [22], this is referred to here as Generalized Lagrangian Path (GLP) approach (or representation) of CQG-theory.

The GLP approach presented here has been shown to be ontologically equivalent to the ”standard” formulation of CQG-theory based on the Eulerian CQG-wave equation. This occurs because, provided the stochastic PDF $f(\Delta g,\hat{g})$ is identified with the Gaussian PDF $\rho_{G}\left(\Delta g\pm \hat{g}\left(r_{o}\right)\right)$ defined above (see Equation (69)), it does not require any kind of addition/modification of the related fundamental axioms established in [10]. This feature permits one to effectively reconcile the Eulerian and Lagrangian descriptions of covariant quantum gravity, which are achieved respectively in terms of the Eulerian and GLP representations of CQG-wave equation and of the quantum wave-function. Nevertheless, it also provides a statistical generalization of the Bohmian interpretation of quantum gravity based on the notion of unique, i.e., deterministic, configuration-space Lagrangian trajectories belonging to the configuration space $U_{g}$ spanned by the symmetric tensor field $g\equiv \left\{g_{\mu \nu }\right\}$. In fact, in the framework of GLP-theory, each Bohmian trajectory is associated with an infinite ensemble of stochastic Lagrangian trajectories associated with the stochastic tensor variable $\Delta g_{\mu \nu }$. Thus, GLP trajectories replace the customary deterministic Lagrangian trajectories (LPs) adopted in the original Bohmian approach, from which they inherently differ for their stochastic character. Consequently, it is shown that it is possible to replace each LP with a corresponding continuum set of stochastic GLP.

A further notable aspect of the GLP approach is, however, that it realizes at the same time also a solution method for the CQG-wave equation and the corresponding equivalent quantum hydrodynamic equations. This is obtained by means of the explicit parameterization of the same equations (and of the quantum wave-function) in terms of the stochastic displacement tensor $\Delta g_{\mu \nu }$ introduced here (see Equation (11)). As an application of the theory developed in this paper, the problem of constructing Gaussian or Gaussian-like solutions of the CQG-wave equation has been addressed. For this purpose, the case of vacuum fields, i.e., obtained in the absence of external classical sources but with the inclusion of a non-vanishing cosmological constant, has been considered. In this connection the explicit construction of solutions of the CQG-quantum hydrodynamic equations has been carried out in which the GLP-parameterized quantum wave function $\psi (G_{L}\left(s\right),\Delta g,s)$ is characterized by a globally-defined Gaussian-like or Gaussian PDF which satisfies identically the corresponding quantum continuity equation. As a notable result, the validity of the emergent-gravity picture has been demonstrated, referred to here as ”*second-type emergent-gravity paradigm*”. Accordingly, the background space-time metric tensor ${\hat{g}}_{\mu \nu }\left(r\right)$ of CQG-theory has been identified in terms of a suitable quantum/stochastic expectation value of the quantum state, i.e., weighted in terms of the corresponding quantum PDF.

In addition, the problem of the construction of separable solutions of the quantum Hamilton-Jacobi (H-J) equation has been posed which satisfy at the same time also the requirements that the quantum wave function $\psi (G_{L}\left(s\right),\Delta g,s)$ is dynamically consistent, in the sense that the corresponding (GLP-parameterized) quantum PDF $\rho (G_{L}\left(s\right),\Delta g,s)$ associated with the quantum wave-function is globally conserved. The solution of the H-J equation has been based on the polynomial representations of the quantum effective potential. In particular, separable solutions for the GLP-parameterized quantum phase function $S(G_{L}\left(s\right),\Delta g,s)$ have been determined based on a harmonic (i.e., second degree) polynomial expansion with respect to the stochastic displacement tensor $\Delta g_{\mu \nu }$. The coefficients of the same expansion have been shown to satisfy an equivalent set of first-order evolution ODEs, denoted as GLP-equations. The same coefficients admit both stationary and non-stationary solutions with respect to the dependence on the background proper-time *s*. Non-stationary solutions include, in particular, the case of small-amplitude solutions which remain globally (i.e., for all *s* greater than the initial proper-time $s_{o}$) suitably close to the stationary ones. These have been identified here with particular solutions exponentially decaying (to the constant ones).

These conclusions show that particular solutions of the CQG-quantum wave-equation exist which are characterized by Gaussian quantum PDF. Remarkably, the same solutions can be either stationary, i.e., characterized by quantum wave-functions of the type $\psi =\psi (G_{L}\left(s\right),\Delta g),$ or non-stationary ones $\psi (G_{L}\left(s\right),\Delta g,s)$, namely depending explicitly on the proper-time *s*. This scenario is promising for its possible implications suggesting that the investigation of non-stationary solutions of the quantum wave-function may be actually an important and challenging subject of future research in quantum gravity, quantum cosmology and CQG-theory.

## Appendix A. Evaluation of *p*(*s*) and Differential Iden-Tities

In this appendix, the proof of Equation (85) in Proposition #4 and the determination of the 4-scalar factor p(s) are explicitly pointed out in the following propositions.

**Proposition** **A1.** **Determination of the tensor field** $\frac{\partial \Delta g_{\alpha \beta }}{\partial g_{L\mu \nu }\left(s^{\prime }\right)}$ *Given validity of the polynomial representation in Equation (83), the tensor field $\frac{\partial \Delta g_{\alpha \beta }}{\partial g_{L\mu \nu }\left(s^{\prime }\right)}$ takes the form*

(A1) $$\frac{\partial \Delta g_{\mu^{\prime }\nu^{\prime }}}{\partial g_{L\mu \nu }\left(s^{\prime }\right)}=-\frac{\partial \Delta g_{\mu^{\prime }\nu^{\prime }}}{\partial G_{L\mu \nu }\left(s^{\prime }\right)},$$

*with*

(A2) $$\frac{\partial \Delta g_{\mu^{\prime }\nu^{\prime }}}{\partial g_{L\mu \nu }\left(s^{\prime }\right)}=\delta_{\mu^{\prime }}^{\mu }\delta_{\nu^{\prime }}^{\nu }p\left(s\right),$$

*and p(s) is the* 4-*scalar function determined by the integral equation*

(A3) $$p\left(s\right)=\frac{1}{1+\int_{s_{o}}^{s}ds^{\prime }\frac{1}{\alpha L}a\left(s^{\prime }\right)g\left(s^{\prime }\right)}.$$

*Here, $a\left(s\right)\equiv \frac{1}{16}a_{\alpha \beta }^{pq}\left(s\right)\delta_{pq}^{\alpha \beta }$ and $a_{\alpha \beta }^{pq}\left(s\right)$ is the tensor introduced in the polynomial decomposition of the phase function $S^{\left(q\right)}$ given by Equation (83)*.

**Proof.** One first notices that, provided the quantum phase function is of the form $S^{\left(q\right)}=S^{\left(q\right)}\left(\Delta g,s^{\prime }\right)$, and noting that $\delta g_{L\mu \nu }^{\left(o\right)}=\delta G_{L\mu \nu }^{\left(o\right)}+\Delta g_{\mu \nu }$, then the LP-initial-value problem in Equation (30) delivers

(A4) $$\delta g_{L\mu \nu }\left(s\right)=\delta g_{L\mu \nu }^{\left(o\right)}+\int_{s_{o}}^{s}ds^{\prime }\frac{1}{\alpha L}\frac{\partial S^{\left(q\right)}\left(\Delta g,s^{\prime }\right)}{\partial g_{L}^{\mu \nu }\left(s^{\prime }\right)},$$

or equivalently

(A5) $$\delta g_{L\mu \nu }\left(s\right)=\delta G_{L\mu \nu }^{\left(o\right)}+\Delta g_{\mu \nu }+\int_{s_{o}}^{s}ds^{\prime }\frac{1}{\alpha L}\frac{\partial S^{\left(q\right)}\left(\Delta g,s^{\prime }\right)}{\partial g_{L}^{\mu \nu }\left(s^{\prime }\right)}.$$

The last equation therefore implies also that the solution to the GLP-initial-value problem in Equation (50) is similarly

(A6) $$\delta G_{L\mu \nu }\left(s\right)=\delta g_{L\mu \nu }\left(s_{o}\right)-\Delta g_{\mu \nu }+\int_{s_{o}}^{s}ds^{\prime }\frac{1}{\alpha L}\frac{\partial S^{\left(q\right)}\left(\Delta g,s^{\prime }\right)}{\partial g_{L}^{\mu \nu }\left(s^{\prime }\right)}.$$

Then, differentiating Equation (A5) with respect to $\delta g_{L\mu \nu }\left(s\right)$ while keeping $\delta G_{L\mu \nu }\left(s_{o}\right)\equiv \delta G_{L\mu \nu }^{\left(o\right)}$ constant, yields

(A7) $$\delta_{\mu^{\prime }}^{\mu }\delta_{\nu^{\prime }}^{\nu }\equiv \frac{\partial g_{L\mu^{\prime }\nu^{\prime }}\left(s\right)}{\partial g_{L\mu \nu }\left(s\right)}=\frac{\partial \Delta g_{\mu^{\prime }\nu^{\prime }}}{\partial g_{L\mu \nu }\left(s\right)}+\frac{\partial \Delta g_{\alpha \beta }}{\partial g_{L\mu \nu }\left(s\right)}\frac{\partial }{\partial \Delta g_{\alpha \beta }}\int_{s_{o}}^{s}ds^{\prime }\frac{1}{\alpha L}\frac{\partial S^{\left(q\right)}\left(\Delta g,s^{\prime }\right)}{\partial g_{L}^{\mu^{\prime }\nu^{\prime }}\left(s^{\prime }\right)},$$

where in the following we shall adopt the short notation $\delta_{\mu^{\prime }\nu^{\prime }}^{\mu \nu }\equiv \delta_{\mu^{\prime }}^{\mu }\delta_{\nu^{\prime }}^{\nu }$ and by construction

(A8) $$\frac{\partial S^{\left(q\right)}\left(\Delta g,s^{\prime }\right)}{\partial G_{L}^{\mu^{\prime }\nu^{\prime }}\left(s^{\prime }\right)}=-\frac{\partial S^{\left(q\right)}\left(\Delta g,s^{\prime }\right)}{\partial g_{L}^{\mu^{\prime }\nu^{\prime }}\left(s^{\prime }\right)},$$

and hence

(A9) $$\frac{\partial \Delta g_{\mu^{\prime }\nu^{\prime }}}{\partial G_{L}^{\mu \nu }\left(s\right)}=-\frac{\partial \Delta g_{\mu^{\prime }\nu^{\prime }}}{\partial g_{L}^{\mu \nu }\left(s\right)}.$$

Consequently, if one performs the differentiation of Equation (A6) with respect to $G_{L\mu \nu }\left(s\right)$ while keeping $\delta g_{L\mu \nu }\left(s_{o}\right)\equiv \delta g_{L\mu \nu }^{\left(o\right)}$ as constant, it follows equivalently that

(A10) $$\delta_{\mu^{\prime }}^{\mu }\delta_{\nu^{\prime }}^{\nu }\equiv \frac{\partial G_{L\mu^{\prime }\nu^{\prime }}\left(s\right)}{\partial G_{L\mu \nu }\left(s\right)}=-\frac{\partial \Delta g_{\mu^{\prime }\nu^{\prime }}}{\partial G_{L\mu \nu }\left(s\right)}-\frac{\partial \Delta g_{\alpha \beta }}{\partial G_{L\mu \nu }\left(s\right)}\frac{\partial }{\partial \Delta g_{\alpha \beta }}\int_{s_{o}}^{s}ds^{\prime }\frac{1}{\alpha L}\frac{\partial S^{\left(q\right)}\left(\Delta g,s^{\prime }\right)}{\partial g_{L}^{\mu^{\prime }\nu^{\prime }}\left(s^{\prime }\right)}.$$

Therefore, from Equation (A7), denoting $\delta_{\mu^{\prime }\nu^{\prime }}^{\mu \nu }\equiv \delta_{\mu^{\prime }}^{\mu }\delta_{\nu^{\prime }}^{\nu }$, it follows

(A11) $$\delta_{\mu \nu }^{\mu^{\prime }\nu^{\prime }}=\frac{\partial \Delta g^{\mu^{\prime }\nu^{\prime }}}{\partial g_{L}^{\mu \nu }\left(s\right)}+\frac{\partial \Delta g^{\alpha \beta }}{\partial g_{L}^{\mu \nu }\left(s\right)}\frac{\partial }{\partial \Delta g^{\alpha \beta }}\int_{s_{o}}^{s}ds^{\prime }\frac{1}{\alpha L}\frac{\partial S^{\left(q\right)}\left(\Delta g,s^{\prime }\right)}{\partial g_{L\mu^{\prime }\nu^{\prime }}\left(s^{\prime }\right)},$$

where due to the polynomial representation in Equation (83)

(A12) $$\begin{array}{c} \left.\frac{\partial S^{\left(q\right)}\left(g_{L}\left(s^{\prime }\right),\Delta g,s^{\prime }\right)}{\partial g_{L\mu^{\prime }\nu^{\prime }}\left(s^{\prime }\right)}=\frac{\partial \Delta g_{pq}}{\partial g_{L\mu^{\prime }\nu^{\prime }}\left(s^{\prime }\right)}\left[a_{p^{\prime }q^{\prime }}^{pq}\left(s^{\prime }\right)\Delta g^{p^{\prime }q^{\prime }}+b^{pq}\left(s\right)\right]\right., \end{array}$$

(A13) $$\begin{array}{c} \left.\frac{\partial }{\partial \Delta g^{\alpha \beta }}\frac{\partial S^{\left(q\right)}\left(g_{L}\left(s^{\prime }\right),\Delta g,s^{\prime }\right)}{\partial g_{L\mu^{\prime }\nu^{\prime }}\left(s^{\prime }\right)}=a_{\alpha \beta }^{\mu \nu }\left(s^{\prime }\right)\frac{\partial \Delta g_{\mu \nu }}{\partial g_{L\mu^{\prime }\nu^{\prime }}\left(s^{\prime }\right)}\right.. \end{array}$$

As a result Equation (A11) delivers

(A14) $$\delta_{\mu \nu }^{\mu^{\prime }\nu^{\prime }}=\frac{\partial \Delta g^{\mu^{\prime }\nu^{\prime }}}{\partial g_{L}^{\mu \nu }\left(s\right)}+\frac{\partial \Delta g^{\alpha \beta }}{\partial g_{L}^{\mu \nu }\left(s\right)}\int_{s_{o}}^{s}ds^{\prime }\frac{a_{\alpha \beta }^{pq}\left(s^{\prime }\right)}{\alpha L}\frac{\partial \Delta g_{pq}}{\partial g_{L\mu^{\prime }\nu^{\prime }}\left(s^{\prime }\right)},$$

thus implying validity of Equation (A2). In fact, thanks to Equation (A2), we can write the previous equation as

(A15) $$\delta_{\mu \nu }^{\mu^{\prime }\nu^{\prime }}=\delta_{\mu \nu }^{\mu^{\prime }\nu^{\prime }}g\left(s\right)+\delta_{\mu \nu }^{\alpha \beta }g\left(s\right)\int_{s_{o}}^{s}ds^{\prime }\frac{a_{\alpha \beta }^{pq}\left(s^{\prime }\right)}{\alpha L}\delta_{pq}^{\mu^{\prime }\nu^{\prime }}g\left(s^{\prime }\right).$$

Then, defining

(A16) $$a\left(s^{\prime }\right)\delta_{\mu \nu }^{\mu^{\prime }\nu^{\prime }}\equiv \delta_{\mu \nu }^{\alpha \beta }a_{\alpha \beta }^{pq}\left(s^{\prime }\right)\delta_{pq}^{\mu^{\prime }\nu^{\prime }}$$

and substituting, after simplification we get that

(A17) $$g\left(s\right)\left[1+\int_{s_{o}}^{s}ds^{\prime }\frac{1}{\alpha L}a\left(s^{\prime }\right)g\left(s^{\prime }\right)\right]=1,$$

while straightforward algebra yields

(A18) $$a\left(s^{\prime }\right)=\frac{1}{16}\delta_{\mu \nu }^{\alpha \beta }a_{\alpha \beta }^{pq}\left(s^{\prime }\right)\delta_{pq}^{\mu^{\prime }\nu^{\prime }}\delta_{\mu^{\prime }\nu^{\prime }}^{\mu \nu }\equiv \frac{1}{16}a_{\alpha \beta }^{pq}\left(s^{\prime }\right)\delta_{pq}^{\alpha \beta }.$$

Thus, provided $1+\int_{s_{o}}^{s}ds^{\prime }\frac{1}{\alpha L}a\left(s^{\prime }\right)g\left(s^{\prime }\right)\neq 0,$ Equation (A3) follows. ☐

**Proposition** **A2.** **Determination of the 4-scalar function** p(s) *In validity of Equation (A3), it follows that*

(A19) $$\left|p(s)\right|=\frac{1}{{\left(1+\frac{2}{\alpha L}\int_{s_{o}}^{s}ds^{\prime }a\left(s^{\prime }\right)\right)}^{1/2}}.$$

**Proof.** In fact, if p(s)≠0, Equation (A17) implies

(A20) $$1+\int_{s_{o}}^{s}ds^{\prime }\frac{1}{\alpha L}a\left(s^{\prime }\right)g\left(s^{\prime }\right)=\frac{1}{p(s)}.$$

Differentiating the same equation term by term with respect to *s* yields the ODE

(A21) $$\frac{1}{\alpha L}a\left(s\right)p\left(s\right)=-\frac{p^{\prime }\left(s\right)}{p^{2}\left(s\right)}.$$

This can be solved noting that $p(s_{o})=1$. Thus, one finds

(A22) $$\frac{1}{2p{\left(s\right)}^{2}}-\frac{1}{2}=\frac{1}{\alpha L}\int_{s_{o}}^{s}ds^{\prime }a\left(s^{\prime }\right),$$

whose solution is given by Equation (A19). ☐

## Appendix B. Differential Identities for the Tensor Coefficients $a_{\mathrm{pq}}^{\alpha \beta }\left(s\right)$

In this appendix, the explicit calculations are reported for several useful identities invoked in Section 7. First, one notices that, invoking Equation (96), it follows that

(A23) $$a_{\mu \nu }^{\alpha \beta }\left(s\right)a_{pq}^{\mu \nu }\left(s\right)=\frac{1}{4}\left[a_{\left(o\right)}^{2}\left(s\right)\delta_{pq}^{\alpha \beta }+\left(4a_{\left(1\right)}^{2}\left(s\right)+2a_{\left(o\right)}\left(s\right)a_{\left(1\right)}\left(s\right)\right){\hat{g}}_{pq}\left(s\right){\hat{g}}^{\alpha \beta }\left(s\right)\right],$$

and similarly

(A24) $$4a_{\left(1\right)}^{2}\left(s\right)+2a_{\left(o\right)}\left(s\right)a_{\left(1\right)}\left(s\right)=2a_{\left(1\right)}^{2}\left(s\right)+4a\left(s\right)a_{\left(1\right)}\left(s\right),$$

(A25) $$\begin{array}{c} \left.a_{\left(o\right)}^{2}\left(s\right)+4a_{\left(1\right)}^{2}\left(s\right)+2a_{\left(o\right)}\left(s\right)a_{\left(1\right)}\left(s\right)=\right.\\ \left.={\left[a_{\left(o\right)}\left(s\right)+a_{\left(1\right)}\left(s\right)\right]}^{2}+3a_{\left(1\right)}^{2}\left(s\right)=4a^{2}\left(s\right)+3a_{\left(1\right)}^{2}\left(s\right).\right. \end{array}$$

The prescription in Equation (96) implies therefore:

(A) from Equation (84):

(A26) $$\begin{array}{ccc} \frac{D}{Ds}S^{\left(q\right)}\left(\Delta g,s\right) & = & \frac{1}{4}\Delta g_{\alpha \beta }\Delta g^{\mu \nu }\left[\frac{d}{ds}a_{\left(o\right)}\left(s\right)\delta_{\mu \nu }^{\alpha \beta }+{\hat{g}}_{\mu \nu }\left(r\right){\hat{g}}^{\alpha \beta }\left(s\right)\frac{d}{ds}a_{\left(1\right)}\left(s\right)\right]\\ & & +\Delta g^{\alpha \beta }\frac{d}{ds}b_{\alpha \beta }\left(s\right)+\frac{d}{ds}c\left(s\right); \end{array}$$

(B) from Equation (89):

(A27) $$\frac{\partial S^{\left(q\right)}\left(G_{L}\left(s\right),\Delta g,s\right)}{\partial g_{L}^{\mu \nu }\left(s\right)}=\frac{1}{2}\left[a_{\left(o\right)}\left(s\right)\delta_{\mu \nu }^{\alpha \beta }+a_{\left(1\right)}\left(s\right){\hat{g}}_{\mu \nu }\left(r\right){\hat{g}}^{\alpha \beta }\left(r\right)\right]\Delta g_{\alpha \beta }p\left(s\right)+b_{\mu \nu }\left(s\right)p\left(s\right).$$

Hence, the quantum 4-tensor fluid velocity field can be represented as

(A28) $$V_{\mu \nu }=\frac{1}{2\alpha L}\left[a_{\left(o\right)}\left(s\right)\delta_{\mu \nu }^{\alpha \beta }+a_{\left(1\right)}\left(s\right){\hat{g}}_{\mu \nu }\left(r\right){\hat{g}}^{\alpha \beta }\left(r\right)\right]\Delta g_{\alpha \beta }p\left(s\right)+\frac{1}{\alpha L}b_{\mu \nu }\left(s\right)p\left(s\right),$$

with the first term on the R.H.S., linearly proportional to Δg, representing the stochastic part of the quantum fluid velocity. Similarly one obtains that, in Equation (89), the following identities hold:

(A29) $$\left\{\begin{array}{c} a_{\mu \nu }^{\alpha \beta }\left(s\right)a_{pq}^{\mu \nu }\left(s\right)=\frac{1}{4}\left[a_{\left(o\right)}^{2}\left(s\right)\delta_{pq}^{\alpha \beta }+\left(4a_{\left(1\right)}^{2}\left(s\right)+2a_{\left(o\right)}\left(s\right)a_{\left(1\right)}\left(s\right)\right){\hat{g}}_{pq}\left(s\right){\hat{g}}^{\alpha \beta }\left(s\right)\right],\\ 2a_{\alpha \beta }^{\mu \nu }\left(s\right)b_{\mu \nu }\left(s\right)=\left[a_{\left(o\right)}\left(s\right)\delta_{\mu \nu }^{\alpha \beta }+a_{\left(1\right)}\left(s\right){\hat{g}}_{\mu \nu }\left(r\right){\hat{g}}^{\alpha \beta }\left(r\right)\right]b^{\mu \nu }\left(s\right). \end{array}\right.$$
